# Hsp90 inhibition increases SOCS3 transcript and regulates migration and cell death in chronic lymphocytic leukemia

**DOI:** 10.18632/oncotarget.8760

**Published:** 2016-04-16

**Authors:** Timothy L. Chen, Nikhil Gupta, Amy Lehman, Amy S. Ruppert, Lianbo Yu, Christopher C. Oakes, Rainer Claus, Christoph Plass, Kami J. Maddocks, Leslie Andritsos, Jeffery A. Jones, David M. Lucas, Amy J. Johnson, John C. Byrd, Erin Hertlein

**Affiliations:** ^1^ Department of Internal Medicine, Division of Hematology, Comprehensive Cancer Center at The Ohio State University, Columbus, Ohio, USA; ^2^ Center for Biostatistics, The Ohio State University, Columbus, Ohio, USA; ^3^ Department of Hematology, Oncology and Stem Cell Transplantation, University Medical Center Freiburg, Freiburg, Germany; ^4^ Division of Epigenomics and Cancer Risk Factors, German Cancer Research Center, Heidelberg, Germany; ^5^ Division of Medicinal Chemistry & Pharmacognosy, College of Pharmacy, The Ohio State University, Columbus, Ohio, USA

**Keywords:** SOCS3, Hsp90, chronic lymphocytic leukemia

## Abstract

Epigenetic or transcriptional silencing of important tumor suppressors has been described to contribute to cell survival and tumorigenesis in chronic lymphocytic leukemia (CLL). Using gene expression microarray analysis, we found that thousands of genes are repressed more than 2-fold in CLL compared to normal B cells; however therapeutic approaches to reverse this have been limited in CLL. Following treatment with the Hsp90 inhibitor 17-DMAG, a significant number of these repressed genes were significantly re-expressed. One of the genes significantly repressed in CLL and up-regulated by 17-DMAG was suppressor of cytokine signaling 3, (SOCS3). SOCS3 has been shown to be silenced in solid tumors as well as myeloid leukemia; however little is known about the regulation in CLL. We found that 17-DMAG induces expression of SOCS3 by via the activation of p38 signaling, and subsequently inhibits AKT and STAT3 phosphorylation resulting in downstream effects on cell migration and survival. We therefore suggest that SOCS3 is an important signaling protein in CLL, and Hsp90 inhibitors represent a novel approach to target transcriptional repression in B cell lymphoproliferative disorders which exhibit a substantial degree of gene repression.

## INTRODUCTION

Chronic lymphocytic leukemia (CLL) is a disease of mature B-cells that accumulate over time due in large part to a resistance to apoptosis. Due to the slowly progressing nature of this disease, studies to determine the molecular events leading to this defective apoptosis are challenging. Our group has previously reported that as disease develops in a CLL mouse model, genes become silenced in a progressive manner [1]. Even though a high degree of methylation is observed in both this mouse model and in CLL patient samples, agents targeting methylation such as decitabine have proven ineffective in CLL therapy [2]. Therefore, other novel methods to reverse gene silencing in CLL are an attractive therapeutic option.

We also observed in the CLL mouse model early transcriptional mechanisms of gene repression involving NF-κB [1]. NF-κB is a family of transcription factors which has been shown to be constitutively active in CLL, typically leading to the transcriptional activation of cell survival and proliferation genes which contribute to disease progression as well as therapeutic resistance [3, 4]. However several studies have also demonstrated that NF-κB plays a prominent role in gene repression as well, either through recruitment of co-repressors [5–7] or through epigenetic silencing [8]. These studies suggest that the inhibition of NF-κB has the potential to increase specific gene targets.

Our lab has previously described the Hsp90 inhibitor 17-DMAG, which is a potent inhibitor of NF-κB signaling in CLL [9]. Hsp90 regulates the stability of proteins involved in multiple signaling pathways that are directly related to CLL cell survival, and we as well as others have previously established Hsp90 inhibition as a promising therapeutic option in CLL [9–13]. Despite this promising preclinical data, Hsp90 inhibitors to date in other cancers have had limited clinical efficacy, in part due to unfavorable side effects associated with treatment. A better understanding of the mechanism should help to refine treatment strategies using Hsp90 inhibitors both as monotherapy and also in combination with other agents. Our earlier work was primarily focused on genes which are activated by NF-κB and subsequently inhibited by 17-DMAG treatment, and did not address the role of 17-DMAG in reversing gene repression. Therefore in the current study we focus on 17-DMAG mediated transcriptional induction or re-expression, and identify suppressor of cytokine signaling 3 (SOCS3). The role of SOCS3 has not been characterized in CLL; however, epigenetic inactivation of SOCS3 has been described to lead to enhanced signaling of survival pathways in solid tumors as well as acute myeloid leukemia (AML)[14–17]. SOCS3 is a negative regulator of the JAK/STAT signaling pathway induced by IL-6, and it's loss could contribute to cell signaling induced by cytokine production from the CLL microenvironment. Therefore re-expression of SOCS3 following treatment with 17-DMAG could represent an important aspect of the cytotoxic mechanism of Hsp90 inhibitors.

## RESULTS

### Gene expression profiling in CLL with 17-DMAG reveals SOCS3 as a repressed target

In this study, we analyzed gene expression in CLL patient samples following treatment with the Hsp90 inhibitor 17-DMAG. We performed expression profiling using the Affymetrix U133 plus 2 array in CLL patient samples (2 pools, each consisting of 5 samples) treated with either vehicle control or 1uM 17-DMAG for 24 hours. We also analyzed expression in B cells isolated from normal donors (1 pool consisting of 6 samples) compared to the CLL samples. We identified 798 probes up-regulated more than 4-fold in CLL samples compared to normal B cells (Supplemental Table 1), while 467 probes were repressed more than 2-fold in CLL samples (Supplemental Table 2). In the current study we have focused on the genes that are repressed in CLL, and in particular how these genes can be therapeutically targeted for re-expression.

**Figure 1:**
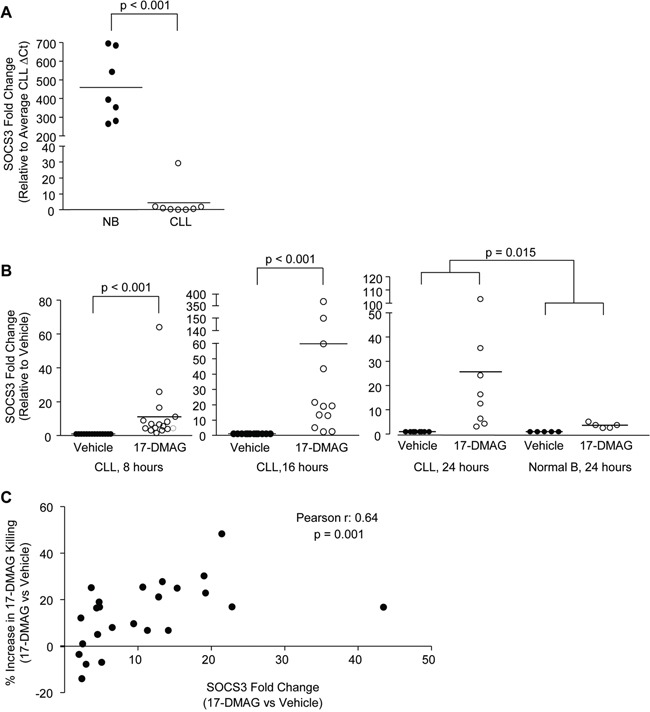
SOCS3 is silenced in CLL and re-expressed following treatment with 17-DMAG **A.** Real time RT-PCR for SOCS3 in normal B cells compared to CLL B cells (N = 7 and N = 8, respectively). Fold change is shown relative to the CLL average expression. **B.** Real time RT-PCR for SOCS3 in CLL B cells treated with vehicle control, or 17-DMAG for 8, 16 and 24 hours (N = 19, N = 14 and N = 8, respectively), or in normal B cells treated with 17-DMAG for 24 hours (N = 5). Data are normalized to TBP transcript and represented as fold change in expression of 17-DMAG treated relative to the vehicle control. Circles represent individual patient samples and the bar represents the average of all patient samples. **C.** Fold change in SOCS3 expression following 17-DMAG treatment of CLL B cells (N = 24) for 16 hours (X-axis, 17-DMAG relative to Vehicle) is compared to 17-DMAG mediated cell killing determined by Ann/PI staining at 24 hours (Y-axis, percent difference in live cells in 17-DMAG relative to Vehicle).

Following treatment with 17-DMAG, a total of 189 probes were decreased more than 4-fold (Supplemental Table 3). Interestingly, although 17-DMAG is known to inhibit multiple transcriptional activators, there were many probes (87) which increased more than 4-fold following treatment (Supplemental Table 4), indicating a potential effect on a transcriptional repressor. Using Ingenuity pathway analysis, we investigated the pathways that are deregulated in CLL cells, both compared to normal B cells as well as following treatment with 17-DMAG. Overall, 345 canonical pathways were deregulated in CLL cells compared to normal B cells and 172 were affected by 17-DMAG treatment. SOCS3 is a critical member of 25 pathways altered in CLL B cells (Table 1), and 22 pathways altered by 17-DMAG treatment (Table 2), and these pathways predominantly involve cytokine signaling. Due to the predominance of SOCS3 in 17-DMAG regulated pathways, and the important role of this gene in cell growth and survival signaling we decided to further explore the mechanism of SOCS3 regulation. We first validated the microarray results using real time RT-PCR and verified that SOCS3 is significantly repressed in CLL compared to normal B cells (Figure 1A, displayed as SOCS3 fold change relative to the CLL average, p < 0.001). Furthermore, *in vitro* treatment with 17-DMAG increased SOCS3 as early as 8 hours (p <0.001) and peaking at 16 hours (p <0.001; Figure 1B). The induction by 24 hours while still significant, is more modest as cells start to undergo apoptosis at this point. Importantly, while 17-DMAG also increased SOCS3 expression in normal B cells at 24 hours, the degree of up-regulation was significantly less than that observed in CLL B cells (Figure 1B, p = 0.015). This is consistent with reduced killing in these cells (compared to CLL B cells) as previously demonstrated by our group [9]. Finally, we found that there was a significant correlation between SOCS3 up-regulation and cell death following 17-DMAG treatment. The samples that had a larger change in viability in the 17-DMAG treated condition relative to the vehicle treated (indicating more cell death) also had higher induction of SOCS3 (Figure 1C; Pearson r = 0.64, p = 0.001). We did not observe an up-regulation of SOCS3 in the B cell leukemia cell lines investigated (697, Mec1) with the exception of the OSU-CLL cell line (derived from CLL patient B cells) recently described by our group [18] (Supplemental Figure 1), indicating that this mechanism may be specific to the primary CLL B cells.

**Table 1:** Ingenuity canonical pathways involving SOCS3: CLL vs NB

#	Ingenuity Canonical Pathways Involving SOCS3: CLL vs NB	Molecules
1	JAK/Stat Signaling	STAT4,SOCS1,**SOCS3**,FOS,JUN,CDKN1A,SOS1,AKT3,JAK2,NFKB2,IL6,NFKB1
2	Type I Diabetes Mellitus Signaling	MAP2K6,**SOCS3**,SOCS1,NFKBIA,HLA-DRB1,CD80,IFNGR1,HSPD1,NFKB2,JAK2,NFKB1,IRF1,BCL2
3	Erythropoietin Signaling	SOCS1,**SOCS3**,FOS,JUN,NFKBIA,SOS1,AKT3,JAK2,NFKB2,NFKB1
4	IL-6 Signaling	MAP2K6,FOS,**SOCS3**,ABCB1,SOCS1,CXCL8,JUN,NFKBIA,SOS1, AKT3,NFKB2,JAK2,IL6,NFKB1
5	IL-10 Signaling	MAP2K6,FCGR2C,**SOCS3**,FOS,JUN,NFKBIA,NFKB2,IL6,FCGR2B, NFKB1
6	3-phosphoinositide Degradation	**SOCS3**,DUSP8,FIG4,STYXL1,PTPN12,DUSP2,MTMR6,CDC25B, INPP5F,TMEM55A,PTPRJ,DUSP1,DUSP10,PTPN22
7	STAT3 Pathway	MYC,SOCS1,**SOCS3**,BMPR1A,TGFBR3,CDKN1A,BMPR2,JAK2, BCL2
8	D-myo-inositol (1,4,5,6)- Tetrakisphosphate Biosynthesis	MTMR6,CDC25B,**SOCS3**,DUSP8,PTPRJ,DUSP1,DUSP10,FIG4, STYXL1,PTPN12,PTPN22,DUSP2
9	D-myo-inositol (3,4,5,6)- tetrakisphosphate Biosynthesis	MTMR6,CDC25B,**SOCS3**,DUSP8,PTPRJ,DUSP1,DUSP10,FIG4, STYXL1,PTPN12,PTPN22,DUSP2
10	D-myo-inositol-5-phosphate Metabolism	**SOCS3**,DUSP8,FIG4,STYXL1,PTPN12,DUSP2,CDC25B,MTMR6, PTPRJ,TMEM55A,DUSP1,DUSP10,PTPN22
11	Role of JAK2 in Hormone-like Cytokine Signaling	SOCS1,**SOCS3**,IRS2,JAK2,HLTF
12	Prolactin Signaling	MYC,SOCS1,**SOCS3**,FOS,JUN,SOS1,JAK2,IRF1
13	Role of JAK1 and JAK3 in γc Cytokine Signaling	BLNK,SOCS1,**SOCS3**,SYK,IL21R,IRS2,JAK2
14	Role of JAK family kinases in IL-6-type Cytokine Signaling	SOCS1,**SOCS3**,JAK2,IL6
15	Acute Phase Response Signaling	MAP2K6,FOS,**SOCS3**,SOCS1,TCF4,JUN,NFKBIA,SOS1,SERPINF1, AKT3,NFKB2,JAK2,IL6,NFKB1
16	Superpathway of Inositol Phosphate Compounds	**SOCS3**,DUSP8,FIG4,STYXL1,INPP5A,PTPN12,DUSP2,MTMR6, CDC25B,INPP5F,TMEM55A,PTPRJ,DUSP1,DUSP10,PTPN22
17	Role of Macrophages, Fibroblasts and Endothelial Cells in Rheumatoid Arthritis	MAP2K6,CXCL8,SOCS1,**SOCS3**,TCF4,ICAM1,WNT3,IL6,JAK2, CREB3L4,NFKB1,MYC,FOS,JUN,NFKBIA,TGFB1,TRAF4,TLR7, AKT3,LEF1,PDGFD
18	3-phosphoinositide Biosynthesis	MTMR6,CDC25B,**SOCS3**,DUSP8,PTPRJ,DUSP1,DUSP10,FIG4, STYXL1,PTPN12,PTPN22,DUSP2
19	IL-9 Signaling	**SOCS3**,IRS2,NFKB2,NFKB1
20	IGF-1 Signaling	SOCS1,**SOCS3**,FOS,JUN,SOS1,AKT3,IRS2,JAK2
21	Type II Diabetes Mellitus Signaling	SOCS1,**SOCS3**,NFKBIA,AKT3,IRS2,NFKB2,NFKB1,ACSL1
22	Growth Hormone Signaling	SOCS1,**SOCS3**,FOS,JAK2,RPS6KA2
23	Insulin Receptor Signaling	**SOCS3**,INPP5F,GAB1,SGK1,SOS1,AKT3,IRS2,JAK2
24	Leptin Signaling in Obesity	**SOCS3**,GNAS,LEPR,AKT3,JAK2
25	IL-22 Signaling	**SOCS3**,AKT3

**Table 2:** Ingenuity canonical pathways involving SOCS3: 17-DMAG vs Vehicle

#	Ingenuity Canonical Pathways Involving SOCS3: DMAG vs Veh	Molecules
1	Type I Diabetes Mellitus Signaling	**SOCS3**,HLA-DRB1,HLA-DMA, HLA-DRA,HLA-DMB,HLA-DQA1,CD86, HLA-DOB,HLA-DQB1,HSPD1
2	D-myo-inositol (1,4,5,6)-Tetrakisphosphate Biosynthesis	**SOCS3**,PTPN6,PPP1R16B,PTPRO,DUSP10
3	D-myo-inositol (3,4,5,6)-tetrakisphosphate Biosynthesis	**SOCS3**,PTPN6,PPP1R16B,PTPRO,DUSP10
4	D-myo-inositol-5-phosphate Metabolism	**SOCS3**,PTPN6,PPP1R16B,PTPRO,DUSP10
5	3-phosphoinositide Degradation	**SOCS3**,PTPN6,PPP1R16B,PTPRO,DUSP10
6	3-phosphoinositide Biosynthesis	**SOCS3**,PTPN6,PPP1R16B,PTPRO,DUSP10
7	Superpathway of Inositol Phosphate Compounds	**SOCS3**,PTPN6,PPP1R16B,PTPRO,DUSP10
8	Role of JAK2 in Hormone-like Cytokine Signaling	**SOCS3**,PTPN6
9	IL-6 Signaling	VEGFA,**SOCS3**,HSPB1
10	Erythropoietin Signaling	**SOCS3**,PTPN6
11	Growth Hormone Signaling	**SOCS3**,PTPN6
12	Role of Macrophages, Fibroblasts and Endothelial Cells in Rheumatoid Arthritis	VEGFA,**SOCS3**,TLR10,TLR7,IGHG1
13	JAK/Stat Signaling	**SOCS3**,PTPN6
14	STAT3 Pathway	**SOCS3**,PTPN6
15	IL-22 Signaling	**SOCS3**
16	Role of JAK family kinases in IL-6-type Cytokine Signaling	**SOCS3**
17	IL-9 Signaling	**SOCS3**
18	Role of JAK1 and JAK3 in γc Cytokine Signaling	**SOCS3**
19	IL-10 Signaling	**SOCS3**
20	Prolactin Signaling	**SOCS3**
21	Leptin Signaling in Obesity	**SOCS3**
22	IGF-1 Signaling	**SOCS3**

Despite the consistent increase in SOCS3 transcript, we were not able to detect a corresponding increase in protein level following 17-DMAG treatment. This is in large part due to the non-specific nature of the SOCS3 antibodies. We tested three different commercially available antibodies for SOCS3, and all three detected a 25 kD band (the predicted size of SOCS3 protein), even in cell lines with undetectable transcript levels of SOCS3 or crispr-mediated deletion of SOCS3 (Supplemental Figure 2). Changes in SOCS3 levels were only detectable in our cell line with super-physiological expression of SOCS3.

### SOCS3 is transcriptionally regulated by 17-DMAG and the p38 pathway

**Figure 2:**
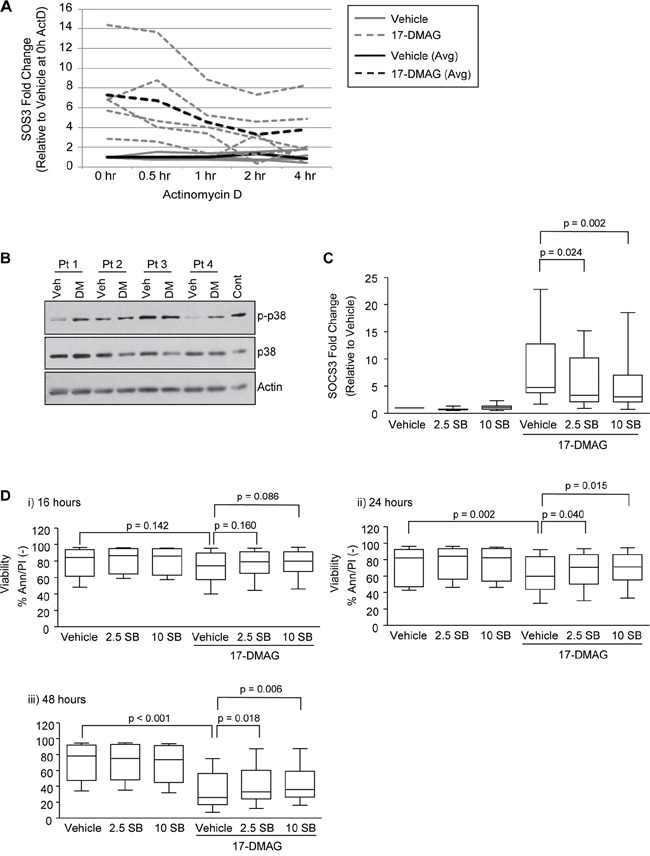
Activation of p38 following treatment with 17-DMAG induces SOCS3 **A.** CLL B cells (N = 5) were treated with vehicle control or 17-DMAG for 16 hours, followed by actinomycin D. RNA was collected at 0, 0.5, 1, 2 and 4 hours and SOCS3 transcript stability was measured by real time RT-PCR. **B.** Immunoblot analysis in representative CLL patient samples (N=4) for phospho-p38 (indicating activation) following 17-DMAG treatment (1 uM) for 16 hours. **C.** Real-time RT-PCR for SOCS3 in CLL B cells treated with 17-DMAG for 16 hours in the presence or absence of SB203580 (2.5 and 10 uM, N = 12). Data are normalized to TBP transcript and represented as fold change in expression of relative to the vehicle only control. **D.** Viability following 17-DMAG treatment for 16 (i), 24 (ii) and 48 (iii) hours in the presence or absence of SB203580 (2.5 and 10 uM, N = 12) determined by Ann/PI staining and flow cytometry.

Given that SOCS3 is silenced by DNA methylation in other malignancies, we first determined if a similar mechanism was acting in CLL cells. Comparison of methylation profiles in the SOCS3 CpG island as well as further upstream regions (Supplemental Figure 3A) revealed no significant differences in CLL DNA methylation relative to normal B cells (Supplemental Figure 3B). Quantitative DNA methylation analysis of the SOCS3 CpG island using the MassARRAY MassCleave assay was performed on two additional regions in the CpG island, one spanning intron 1 and exon 2 just upstream of the translational start site of SOCS3 (SOCS3-ATG; 31 CpGs analyzed) the other 5′ of the TSS (SOCS3-5′; 15 CpGs analyzed). No difference in DNA methylation between CLL and normal B cells was found (Supplemental Figure 3C). This suggests that SOCS3 is transcriptionally silenced by a mechanism other than methylation. In order to determine whether SOCS3 was induced in response to 17-DMAG rather than 17-DMAG providing enhanced transcript stability, we treated CLL cells with 17-DMAG for 16 hours followed by the addition of actinomycin D to inhibit new transcription. SOCS3 transcript decay was then monitored up to 4 hours. As expected, SOCS3 transcript is significantly induced by 17-DMAG (Figure 2A, 17-DMAG ActD-0hr vs. Vehicle ActD-0hr, p <.001). However, in the presence of actinomycin D, there is no significant difference in SOCS3 transcript stability over 4 hours (Figure 2A, 17-DMAG ActD-4hr vs 17-DMAG time ActD-0hr, p = 0.481). These results indicate a mechanism of transcriptional activation versus transcript stabilization.

In our microarray data, FosL2 and JunD were also significantly repressed in CLL cells compared to normal B cells, and were up-regulated following 17-DMAG treatment (Supplemental Tables 2 and 4). The p38 MAPK pathway directly regulates Fos family genes [19], and several studies have indicated that the p38 pathway can also regulate SOCS3 transcript [20–22]. Finally, several CLL therapies (flavopiridol, rituximab and OSU-DY7) have been shown to induce p38 dependent apoptosis in CLL [23–25]. Therefore we hypothesized that p38 signaling was induced by 17-DMAG in CLL, and responsible for the increase in SOCS3 transcript and subsequent cell death. We treated primary CLL cells with 17-DMAG for 16, 24 and 48 hours in the presence or absence of the p38/MAPK pathway inhibitor SB203580 (both 2.5 and 10 uM). We found that 17-DMAG does in fact induce p38 activity, indicated by increased phosphorylation of p38 (Figure 2B). Furthermore, treatment with SB203580 at either dose significantly inhibited SOCS3 up-regulation following 17-DMAG treatment (Figure 2C; p = 0.024 for 2.5 uM SB, and p = 0.002 for 10uM SB at 16 hours). Additionally, we found that treatment with SB203580 impaired the ability of the Hsp90 inhibitor to kill the CLL cells (Figure 2Dii; p = 0.040 for 2.5 uM SB, and p = 0.015 for 10uM SB at 24 hours, and Figure 2Diii; p = 0.018 for 2.5 uM SB, and p = 0.006 for 10uM SB at 48 hours). 17-DMAG had no cytotoxic effect on the cells at 16 hours (p = 0.142, Figure 2Di) and the p38 inhibitor therefore showed no significant difference in 17-DMAG mediated killing, which is consistent with our previous data that Hsp90 inhibition does not initiate significant cell death prior to 24 hours.

### Re-expression of SOCS3 inhibits IL-6 and SDF-1 induced signaling and migration

**Figure 3:**
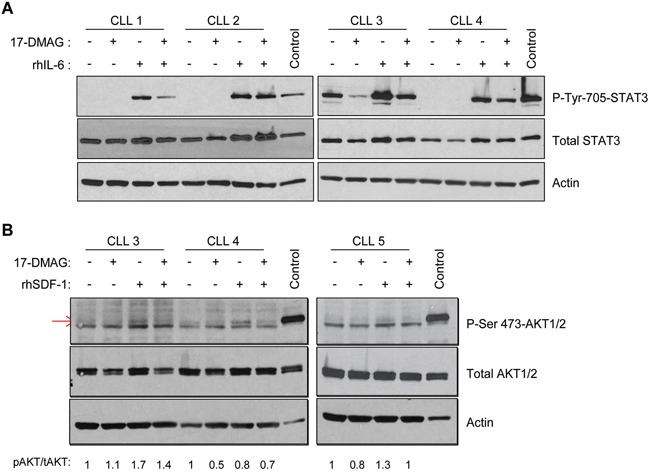
17-DMAG inhibits IL-6 and SDF-1 induced signaling **A.** CLL B cells were treated with vehicle control or 17-DMAG for 8 hours, followed by stimulation with recombinant human IL-6 (rhIL-6). Immunoblots were performed for p-STAT3 and total STAT3, as well as a loading control actin. Results shown are representative of 12 patient samples. **B.** CLL B cells were treated with vehicle control or 17-DMAG for 8 hours, followed by stimulation with recombinant human SDF-1 (rhSDF-1). Immunoblots were performed for p-AKT and total AKT, as well as a loading control Actin. Densitometry for p-AKT (indicated by the red arrow) relative to total AKT (each protein first normalized to actin) is indicated below the blots. Results shown are representative of 6 patient samples.

SOCS3 can disrupt the SDF-1/CXCR4 pathway [26–28] as well as signaling to either focal adhesion kinase (FAK) or AKT which are both key survival factors induced by IL-6 in CLL. Therefore in order to determine the downstream effects of SOCS3 regulation in CLL cells, we analyzed the effect of IL-6 and SDF-1 stimulation. CLL cells were treated with 17-DMAG for 8 hours (the earliest time point of SOCS3 induction without any evidence of cell death), followed by stimulation with recombinant human IL-6 (rhIL-6). Phosphorylation of STAT3 (Tyr705) was induced following rhIL-6 stimulation, and this effect was substantially blocked with 17-DMAG pre-treatment (Figure 3A). This occurred prior to degradation of total STAT3 protein, which is ultimately expected due to loss of the chaperone function provided by Hsp90 [29]. When 17-DMAG and IL-6 are added concurrently, p-STAT3 is still reduced, albeit less than pre-treatment with 17-DMAG (data not shown). This is again consistent with the time required for SOCS3 induction rather than an immediate direct effect of 17-DMAG on STAT3 phosphorylation. In a manner similar to IL-6, we found that an 8 hour pre-treatment with 17-DMAG is able to block the SDF-1 induced phosphorylation of AKT as well (Figure 3B, induced p-AKT is the upper band indicated by the red arrow). Densitometry for p-AKT relative to total AKT (each protein first normalized to actin) is shown below the blots. Jurkat T cell lysate was used as a positive control as these cells have been shown to have high levels of constitutive p-AKT.

**Figure 4:**
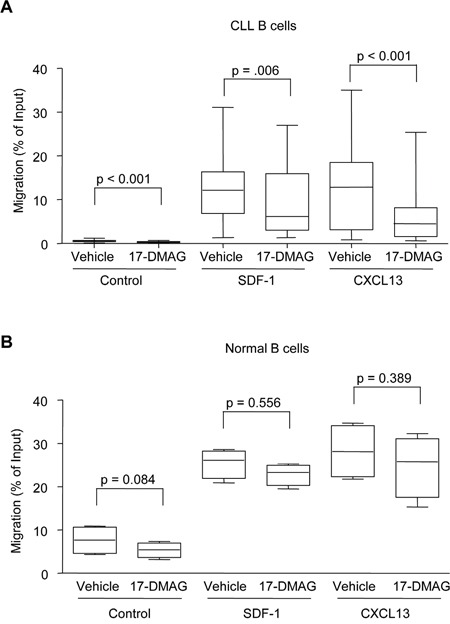
17-DMAG and re-expression of SOCS3 inhibits migration **A.** CLL B cells (N = 14 for CXCL13, N = 16 for SDF-1) were re-suspended at 5 × 10^6^ cells/mL and treated with vehicle control or 17-DMAG for 5 hours, then were placed in the upper well of 24-well transwell plates. The bottom wells contained either media alone, or media with recombinant SDF-1 (200 ng/mL) or CXCL13 (1000 ng/mL). Cells in the lower chamber were collected after 3 additional hours (for a total of 8 hours 17-DMAG treatment), and percent migration is calculated relative to the input. **B.** Normal B cells (N = 4) were re-suspended at 5 × 10^6^ cells/mL and treated with vehicle control or 17-DMAG for 5 hours, then were placed in the upper well of 24-well transwell plates. The bottom wells contained either media alone, or media with recombinant SDF-1 (200 ng/mL) or CXCL13 (1000 ng/mL). Cells in the lower chamber were collected after 3 additional hours (for a total of 8 hours 17-DMAG treatment), and percent migration is calculated relative to the input.

Given this effect on SDF-1 signaling, we next sought to determine whether this pathway was functionally inhibited following Hsp90 treatment using *in vitro* cell migration assays. Pre-treatment of primary CLL cells with 17-DMAG significantly inhibited the migration towards both SDF-1 (p = 0.006) and CXCL13 (p < 0.001) (Figure 4A). Interestingly, even though very few cells migrated towards the control media with no chemokine, 17-DMAG still had a significant effect on migration (p < 0.001) indicating that inhibition of Hsp90 plays a role in the overall motility of the CLL cells. Finally, under the same conditions we determined the effect of 17-DMAG on the migration of normal B cells. While these cells were able to efficiently migrate towards chemokine (even more than the CLL B cells), 17-DMAG was not able to significantly inhibit the migration of these cells towards SDF-1 (p = 0.556) or CXCL13 (p = 0.389) (Figure 4B), which is consistent with the real time data showing less induction of SOCS3 in normal B cells.

### Exogenous expression of SOCS3 in a B cell line inhibits IL-6 and SDF-1 induced signaling

**Figure 5:**
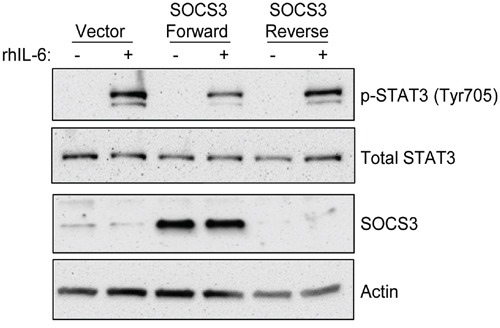
Re-expression of SOCS3 inhibits IL-6 signaling in a CLL cell line The OSU-CLL B cell line was modified to over-express the SOCS3 coding sequence. Cell lines expressing the coding sequence in the reverse orientation or the empty vector only were used as controls. Immunoblots were performed for p-STAT3, total STAT3, and SOCS3, as well as a loading control Actin.

Finally, in order to verify the specific role of SOCS3 on these signaling pathways, we utilized a CLL B-cell line previously described by our lab (OSU-CLL) to over-express SOCS3. This cell line was chosen for mechanistic studies as it is the only line where SOCS3 induction with 17-DMAG is evident, and unlike other CLL cell lines, OSU-CLL responds to IL-6 induction. As shown in Figure 5, exogenous over-expression of SOCS3 is able to reduce the phosphorylation of STAT3 following stimulation with IL-6, whereas the control vector or SOCS3 expressed in the reverse orientation had no effect on this signaling. These data suggest that SOCS3 is able to regulate cell signaling in a CLL B-cell line.

## DISCUSSION

The regulation of SOCS3 has not been implicated in CLL. In this study, we have provided evidence that SOCS3 can regulate important cell survival pathways in CLL. The correlation between up-regulation of SOCS3 and cell death in primary CLL cells indicate that this is an important factor in the mechanism of Hsp90 inhibitor mediated cell death.

We have provided evidence that the regulation of SOCS3 in CLL is not due to silencing via DNA methylation, but rather our data does support a role for p38 signaling in SOCS3 transcriptional regulation. In general, p38 signaling is reported to induce cell survival in CLL cells, likely due to crosstalk with the tumor microenvironment [30]. However, some studies have shown that CD40L induced p38 is actually reduced in CLL cells compared to normal B cells [31], and our array data suggest that some of the downstream substrates of this pathway (Jun/Fos transcription factors) are also silenced in CLL. Therefore while p38 signaling may have dual roles in CLL, we have demonstrated here that it is involved in SOCS3 up-regulation and the cytotoxic mechanism of 17-DMAG treatment.

SOCS3 is predominantly considered a negative regulator of the IL-6 induced JAK/STAT pathway which can be constitutively active in CLL due to interaction with other cells in the tumor microenvironment, or due to treatment related complications such as cytokine release following tumor lysis. In addition to IL-6, the T cell marker CD5, which is aberrantly expressed on CLL B-cells, has been shown to signal through STAT3 to activate cytokine production in CLL [32]. Another signaling pathway regulated by SOCS3 and particularly important to the migration and survival of CLL cells is the SDF-1/CXCR4 pathway. SDF-1 signaling in B cells has been shown to induce phosphorylation of BTK and downstream PLCγ2, which is in turn responsible for B cell migration [33]. Phosphoproteomic analysis identified several other targets downstream of SDF-1 signaling associated with CLL cell survival, including Hsp90 [34]. Finally, drugs such as the tyrosine kinase inhibitor dasatinib have been shown to inhibit CLL cell migration and signaling to AKT by blocking SDF-1 signaling [35].

Given the documented importance of these signaling pathways in the survival of CLL cells, the ability to therapeutically target these pathways simultaneously would be advantageous in the treatment of CLL. Similar to what has been demonstrated in other cell types, our data show that 17-DMAG impaired both the phosphorylation of STAT3 following IL-6 stimulation as well as AKT phosphorylation following SDF-1 stimulation. 17-DMAG also reduced the ability of CLL cells to migrate *in vitro* towards chemokine. Consistent with what has been previously reported, the normal B cells migrated more than CLL both towards media alone or media containing chemokine [34]. However, 17-DMAG did not up-regulate SOCS3 in normal B cells to the same extent as CLL B cells (Figure 1C), and also did not inhibit the migration of normal B cells (Figure 4B). These results highlight the importance of this up-regulation as a tumor-specific mechanism.

In conclusion, we have demonstrated that up-regulation of SOCS3 via the p38 pathway is involved in the mechanism of Hsp90 inhibitor induced CLL cell death. Higher SOCS3 levels have been shown to be associated with the lack of bulky lymphadenopathy and splenomegaly in CLL [36]. However, this work provides the first evidence of the mechanism of SOCS3 regulation in CLL, and uncovers a potentially new therapeutic target in this disease. We have demonstrated this using the Hsp90 inhibitor 17-DMAG, however the role of SOCS3 independent of Hsp90 inhibitors should also be explored. This could include a screen for other inhibitors that more specifically regulate SOCS3, or potentially the development of a small molecule SOCS3 mimetic. Due to the documented role of SOCS3 silencing in other types of cancer, this type of therapeutic intervention could be applicable to the treatment of acute leukemia and solid tumors as well.

## MATERIALS AND METHODS

### Patients, cell separation, culture conditions, and reagents

For *in vitro* studies, written, informed consent was obtained to procure cells from patients with previously diagnosed CLL as defined by the modified NCI criteria [37]. Mononuclear cells from CLL patients and normal volunteers were isolated and placed in culture as previously described by our group [9]. Cells were grown in RPMI media with 10% fetal bovine serum, l-glutamine and antibiotics. No other additives were included with the exception of cell migration experiments where recombinant SDF-1 and CXCL13 were added to the media. 17-DMAG was obtained from the Division of Cancer Treatment and Diagnosis, National Cancer Institute (Bethesda, MD).

### Microarray analysis

Gene expression were analyzed by Affymetrix Expression Console software. RMA method was used to do background correction, normalization and probe set summarization. A filtering method based on percentage of arrays above noise cutoff was applied to filter out low expression genes. Microarray results are available through GEO entry #GSE76546.

### Viability assays

Apoptosis was determined by staining with annexin V-FITC and propidium iodide (PI). After exposure to 1 uM 17-DMAG, cells were stained in 1X binding buffer (BD Biosciences, San Jose, CA). Cell death was assessed by flow cytometry using a Beckman-Coulter cytometer (Beckman-Coulter, Miami, FL). Ten thousand cells were counted for each sample, and data was analyzed with the Kaluza software package (Beckman-Coulter).

### Cell migration assays

Cells were pretreated with 1 uM 17-DMAG for 5 hours. Cells (5×10^6^ cells/mL) were then transferred to the upper chamber of a 24-transwell plate with a 5μm filter. Chambers were placed into wells containing media with either no chemokine (control), recombinant human SDF-1 (200 ug/mL) or CXCL13 (1000 ng/mL). Migration was permitted for 3 hours, and cells in the lower chamber were collected and counted for 20 seconds on high speed on a Beckman Coulter FC500 flow cytometer. A 1/20 dilution of input cells was also determined (input).

### Immunoblot analysis

Antibodies used for immunoblots included AKT, p-AKT, STAT3, p-STAT3, p38 and p-p38 (Cell Signaling, Danvers, MA); SOCS3 (Abcam, Cambridge, MA; Cell Signaling and Santa Cruz Biotechnology, Santa Cruz, CA); and actin (Santa Cruz). Protein (50 μg/lane) was separated on polyacrylamide gels and transferred onto nitrocellulose. Following antibody incubations, proteins were detected with chemiluminescent substrate (Pierce) and quantified using a ChemiDoc system with Quantity One software (Bio-Rad Laboratories, Hercules, CA).

### Real-time RT-PCR

RNA was extracted by phenol chloroform isolation using TRIzol reagent (Invitrogen, Grand Island, NY) and purified using Qiagen RNeasy columns (Qiagen, Valencia, CA). cDNA was prepared with SuperScript First-Strand Synthesis System for RT-PCR (Invitrogen). Real-time polymerase chain reaction was performed using commercially available primers (Applied Biosystems, Foster City, CA). Detection was performed using an ABI Prism 7700 sequence detection system (Applied Biosystems). Average relative expression (treatment compared to media) was normalized to the internal control genes TBP or 18S.

### Statistical analysis

Linear mixed effects models were used to assess differences between conditions of interest for experiments involving RT-PCR data (ΔCT values; Figure 1A, 1B, and 2B), viability (% Ann/PI negative cells; Figure 2C), and migration (% of input, log- transformed; Figure 4). For Figure 1C, the Pearson correlation was calculated to assess the linear relationship between the % change in viability and SOCS3 fold change (log_2_-transformed values). All analyses were performed using SAS/STAT software version 9.3 (SAS Institute, Inc., Cary, NC).

## SUPPLEMENTARY FIGURES AND TABLES










