# Supplementary material for: Hsp90 inhibition increases SOCS3 transcript and regulates migration and cell death in chronic lymphocytic leukemia
Source: Oncotarget. 2016 Apr 16;7(19):28684–96. doi: 10.18632/oncotarget.8760 (PMC5053755; doi:10.18632/oncotarget.8760)
Supplement: Supplementary file 1 [file oncotarget-07-28684-s001.pdf]

Hsp90 inhibition increases SOCS3 transcript and regulates migration and cell death in chronic lymphocytic leukemia

SUPPLEMENTARY FIGURES AND TABLES

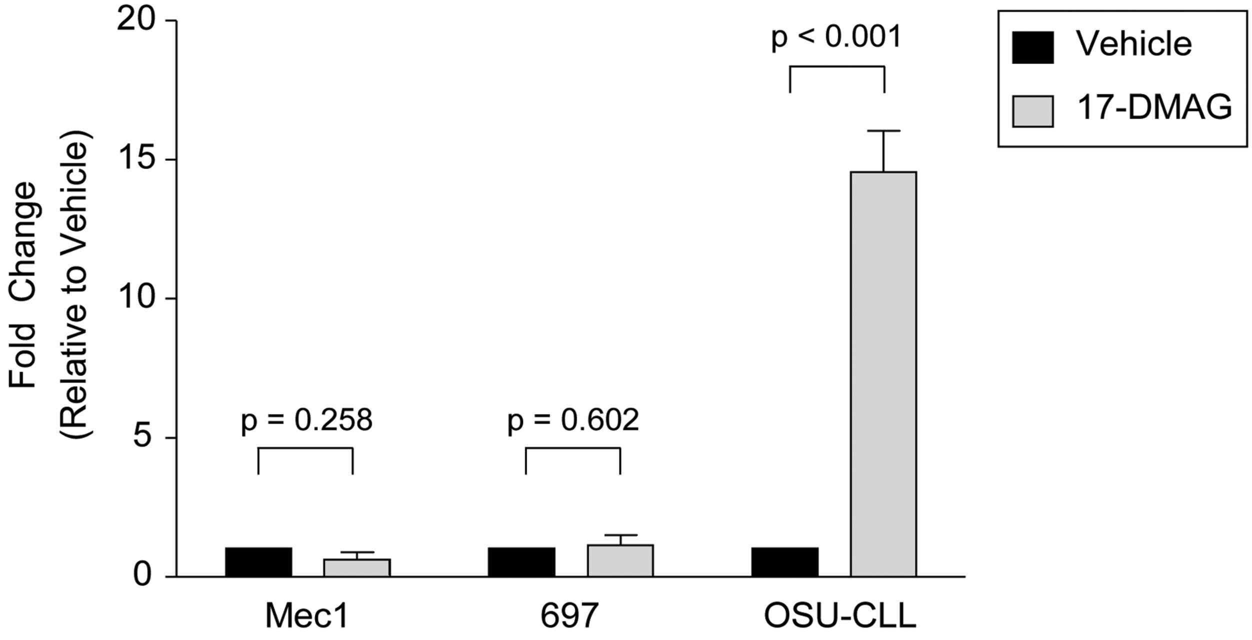

Supplementary Figure S1: SOCS3 transcriptional regulation by 17-DMAG in B cell lines. Real time RT-PCR for SOCS3 in B cell lines treated with vehicle control or 17-DMAG for 24 hours (N = 3 for 697 and Mec1, N = 5 for OSU-CLL). Data are normalized to TBP transcript and represented as fold change in expression of 17-DMAG treated relative to the vehicle control.

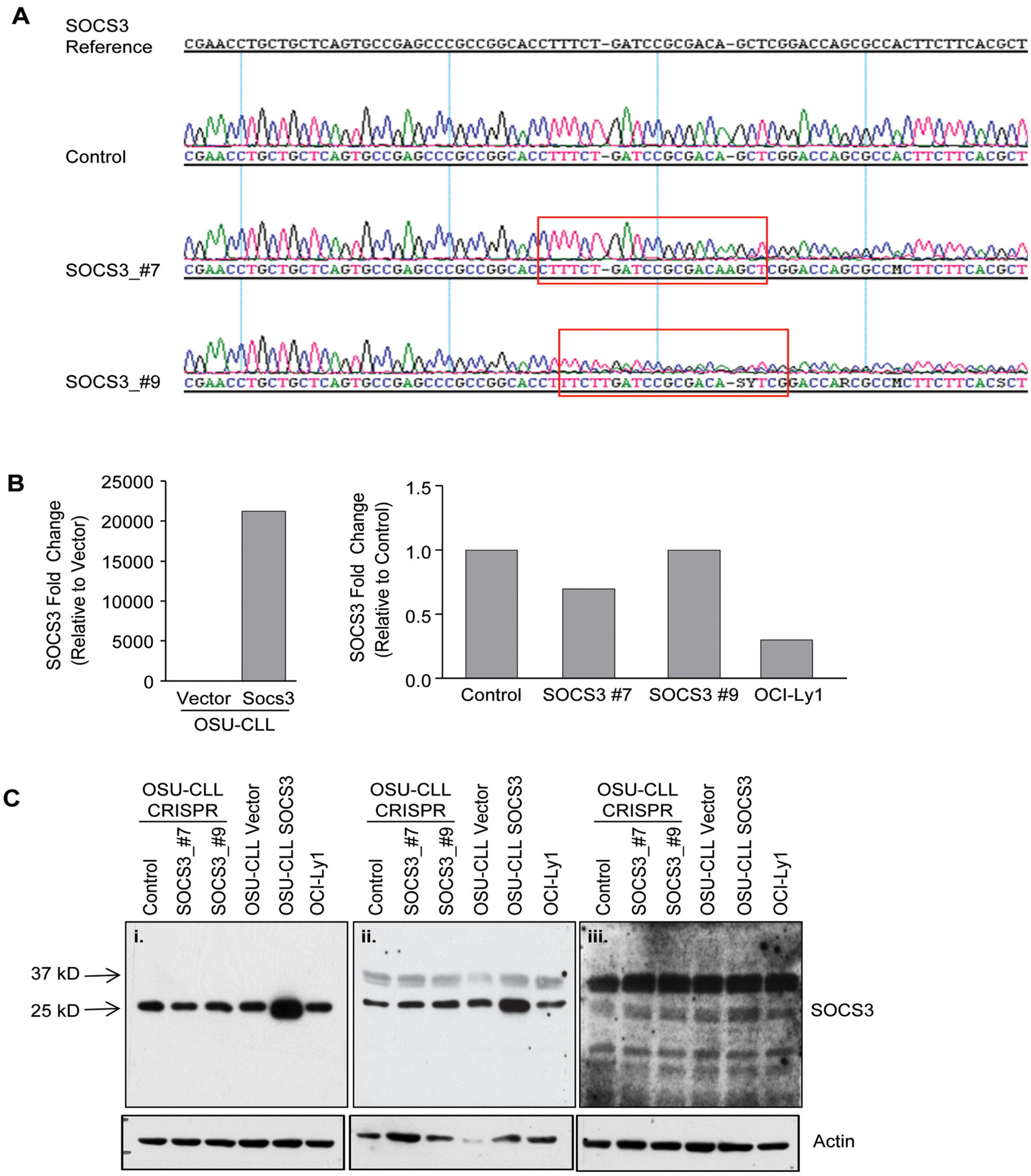

Supplementary Figure S2: Crispr mediated deletion of SOCS3. A. Two different guide RNAs (#7 and #9) as well as a non-targeting control were used to target the SOCS3 genomic locus in a CLL B cell line (OSU-CLL). The specific regions recognized by the guide RNAs are indicated by the red box. The effect of the crispr editing at the level of genomic DNA was verified by amplifying the targeted region using PCR followed by Sanger sequencing. Random 1-2 base pair insertions or deletions result in disrupted genomic sequence downstream of the guide RNA binding site. **B.** Real time RT-PCR for SOCS3 in OSU-CLL Vector and SOCS3 expressing cell lines, crispr edited cell lines, and OCI-Ly1. **C.** Immunoblots were performed using SOCS3 antibodies from 3 different sources. The OSU-CLL B cell line has been modified using CRISPR to delete SOCS3 (lanes 1-3), or to over-express SOCS3 (lane 5). Also included as a negative control is the OCI-Ly1 lymphoma cell line (lane 6), which has no detectable SOCS3 transcript (Ct > 40). Antibodies are as follows: **i.** Abcam (ab 16030). **ii.** Santa Cruz (SC-7009). **iii.** Cell Signaling (2923S).

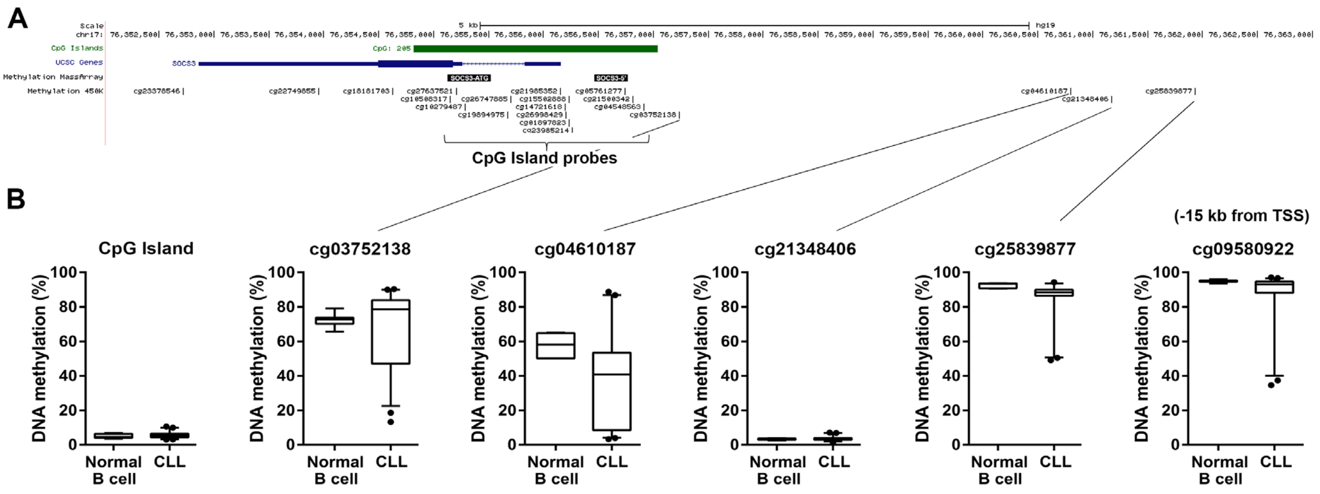

C

DNA methylation analysis using MassARRAY:

Amplicon	# CpGs in amplicon	Average methylation Normal B (N=6)	CLL (N=99)
SOCS3-5'	15	7.0%	6.0%
SOCS3-ATG	31	8.0%	8.0%

Supplementary Figure S3: SOCS3 methylation in CLL and normal B cells. DNA methylation analysis of the SOCS3 promoter and 5' upstream region using MassARRAY and Illumina 450K arrays. **A.** Diagram of the SOCS3 locus showing the SOCS3 gene (blue) and associated promoter CpG island (green) along with the positions of MassARRAY amplicons (black) and Illumina 450K probes. The SOCS3 gene is oriented right-to-left (opposite strand). Figure obtained from the UCSC Genome Browser (https://genome.ucsc.edu, GRCh37/hg19 Assembly). **B.** DNA methylation levels of 450K probes in CD19+ sorted normal B cells (n=7) and CLL (n=128). Data displayed using box-and-whisker plots (line represents median, bars 2.5-97.5 percentile) and obtained from Oakes et al. (PMID: 26780610); available at the European Genome-Phenome Archive (https://ega-archive.org), accession EGAS00001000534. All fourteen 450K probes contained within the CpG island sequence were averaged per sample due to their uniformly low methylation levels. Probes upstream of the CpG island are displayed individually, including an additional probe (cg09580922) located 15 kb upstream of the SOCS3 translational start site (TSS). No significant increase in CLL DNA methylation levels relative to normal B cells was found. **C.** Quantitative DNA methylation analysis of the SOCS3 CpG island using the MassARRAY MassCleave assay (Agena Biosciences) in CD19+ sorted normal B cells (n=6) and CLL (n=99). Two amplicons within the SOCS3 CpG island were analyzed, one spanning intron 1 and exon 2 just upstream of the translational start site of SOCS3 (SOCS3-ATG; 31 CpGs analyzed) the other 5' of the TSS (SOCS3-5'; 15 CpGs analyzed). CpGs were averaged per amplicon. No difference in DNA methylation between CLL and normal B cells was found.

Supplementary Table S1: Probe sets up-regulated 4-fold or greater in CLL vs NB cells

See Supplementary File 1

Supplementary Table S2: Probe sets down-regulated 4-fold or greater in CLL vs NB cells

See Supplementary File 2

Supplementary Table S3: Probe sets down-regulated 4-fold or greater in DMAG treated vs Vehicle

See Supplementary File 3

Supplementary Table S4: Probe sets up-regulated 4-fold or greater in DMAG treated vs Vehicle

See Supplementary File 4
