# Supplementary material for: Hsp90 inhibition increases SOCS3 transcript and regulates migration and cell death in chronic lymphocytic leukemia
Source: Oncotarget. 2016 Apr 16;7(19):28684–96. doi: 10.18632/oncotarget.8760 (PMC5053755; doi:10.18632/oncotarget.8760)
Supplement: Supplementary file 2 [file oncotarget-07-28684-s002.docx]

Supplemental Table 1: Probe sets up-regulated 4-fold or greater in CLL vs NB cells

# Probe fold:CLL/Normal B

1 217504_at 151.4973765

2 229070_at 147.8540549

3 221558_s_at 81.70712589

4 209598_at 81.4613885

5 1553708_at 78.03005436

6 236341_at 58.84019

7 242344_at 57.82722633

8 205414_s_at 45.86071683

9 205466_s_at 42.94400978

10 1562587_at 40.46111017

11 238323_at 39.1256957

12 229103_at 38.45356632

13 209470_s_at 36.11266537

14 202709_at 32.74818397

15 226147_s_at 26.43362722

16 203608_at 25.9940045

17 215100_at 25.52500786

18 210948_s_at 24.22655728

19 209469_at 23.79319322

20 203232_s_at 23.6976238

21 210972_x_at 22.88253528

22 235401_s_at 22.30442421

23 230793_at 22.01302489

24 204072_s_at 20.50348156

25 230109_at 19.37481326

26 213374_x_at 19.36700552

27 266_s_at 18.93486198

28 209392_at 18.82898726

29 225045_at 18.38564647

30 212136_at 18.32188457

31 211902_x_at 18.11159879

32 213388_at 17.85851853

33 228190_at 17.72634264

34 239185_at 17.68067563

35 209670_at 17.62526446

36 230551_at 17.56569191

37 212698_s_at 17.54300515

38 219840_s_at 17.27439166

39 203231_s_at 16.73049818

40 201506_at 16.65548546

41 242541_at 16.64158567

42 1570239_a_at 16.58666575

43 234440_at 16.56408952

44 235400_at 16.32731889

45 232821_at 16.04900554

46 209671_x_at 16.01324736

47 238462_at 15.96178952

48 1555882_at 15.91742432

49 205698_s_at 15.8419717

50 219304_s_at 15.80516838

51 232533_at 15.68486819

52 216379_x_at 15.6836234

53 204890_s_at 15.50486973

54 204891_s_at 15.48146399

55 220007_at 15.35222122

56 238593_at 15.23675736

57 235372_at 15.18476841

58 208651_x_at 15.04654138

59 1562754_at 14.91716011

60 234362_s_at 14.84826028

61 230489_at 14.78116933

62 240413_at 14.7509299

63 200762_at 14.60571926

64 209512_at 14.58575843

65 205234_at 14.45013434

66 203548_s_at 14.34482537

67 209771_x_at 14.09725143

68 1554667_s_at 14.07877593

69 226408_at 14.01183015

70 215767_at 13.91971242

71 239233_at 13.80004437

72 225207_at 13.73187286

73 219955_at 13.67483481

74 238601_at 13.62875621

75 225540_at 13.61798175

76 221556_at 13.24914172

77 209513_s_at 13.2453678

78 221337_s_at 13.15225253

79 205352_at 12.84770707

80 230252_at 12.79652338

81 225989_at 12.63599179

82 232060_at 12.45090755

83 237515_at 12.34963502

84 213566_at 12.20653649

85 203711_s_at 12.14325262

86 231472_at 12.13634838

87 219387_at 11.8943003

88 209772_s_at 11.87592515

89 226884_at 11.86126931

90 229114_at 11.75929366

91 1556051_a_at 11.6510898

92 1556474_a_at 11.4793894

93 202054_s_at 11.43707715

94 209995_s_at 11.28332217

95 226685_at 11.19667916

96 39318_at 11.1777817

97 204563_at 10.91196487

98 213005_s_at 10.76706774

99 241808_at 10.70330173

100 1562648_at 10.69492529

101 234980_at 10.6610786

102 205552_s_at 10.65776114

103 204199_at 10.55360509

104 225639_at 10.48759519

105 208650_s_at 10.4537235

106 230875_s_at 10.38649872

107 213578_at 10.38169785

108 221331_x_at 10.309168

109 221030_s_at 10.29492918

110 221078_s_at 10.28653008

111 224496_s_at 10.20376378

112 222819_at 10.16979012

113 231794_at 10.15110236

114 221455_s_at 10.08158802

115 218477_at 10.08025339

116 219301_s_at 9.993641739

117 227834_at 9.926282403

118 205805_s_at 9.862586169

119 224368_s_at 9.859517182

120 47553_at 9.823911064

121 206170_at 9.725321881

122 204832_s_at 9.703959303

123 206150_at 9.604929336

124 226649_at 9.570491385

125 242584_at 9.522933116

126 201413_at 9.462208821

127 203178_at 9.460661094

128 209498_at 9.430991811

129 201525_at 9.40795722

130 228377_at 9.300008276

131 1558185_at 9.241185892

132 238587_at 9.201812119

133 218983_at 9.174582449

134 207417_s_at 9.116597616

135 210889_s_at 9.091239469

136 226126_at 8.938907388

137 205699_at 8.807295063

138 202421_at 8.663223876

139 242247_at 8.648041822

140 225385_s_at 8.628546175

141 235762_at 8.580918013

142 224666_at 8.511285232

143 203264_s_at 8.490897166

144 204361_s_at 8.466780571

145 206857_s_at 8.402589474

146 226591_at 8.391164283

147 1552497_a_at 8.357833393

148 204438_at 8.348401582

149 218968_s_at 8.346691796

150 221908_at 8.326409424

151 229053_at 8.263282554

152 1555883_s_at 8.22929876

153 237411_at 8.218280088

154 206372_at 8.203147104

155 230777_s_at 8.187519854

156 227396_at 8.175588028

157 217950_at 8.142128554

158 207583_at 8.115899499

159 203143_s_at 8.09687946

160 231166_at 8.090778352

161 210839_s_at 8.08037641

162 219452_at 8.079441116

163 227607_at 8.052506946

164 202382_s_at 8.044961406

165 203685_at 8.044479067

166 222453_at 8.035520626

167 223588_at 8.033384892

168 38671_at 8.0293127

169 225806_at 7.982991639

170 242248_at 7.948369822

171 212345_s_at 7.94661803

172 228245_s_at 7.935950351

173 219654_at 7.899796556

174 225341_at 7.889569059

175 229075_at 7.844614217

176 219048_at 7.839086257

177 214806_at 7.828842393

178 200799_at 7.777155042

179 229133_s_at 7.775926056

180 230866_at 7.772075954

181 227312_at 7.735137836

182 230287_at 7.715368157

183 226002_at 7.677656419

184 238649_at 7.676381967

185 219008_at 7.653936861

186 210550_s_at 7.64564916

187 57082_at 7.616918716

188 208438_s_at 7.564165872

189 202661_at 7.53381183

190 226517_at 7.533302699

191 220180_at 7.524399765

192 243539_at 7.515400561

193 225285_at 7.508868362

194 222428_s_at 7.505236324

195 203609_s_at 7.501866034

196 221589_s_at 7.494010505

197 222108_at 7.479892313

198 208022_s_at 7.475929686

199 206513_at 7.450299899

200 223177_at 7.448386826

201 225496_s_at 7.44518398

202 210617_at 7.420179052

203 213714_at 7.412026298

204 212235_at 7.40752197

205 231647_s_at 7.383641464

206 214318_s_at 7.360450144

207 214131_at 7.336291683

208 238452_at 7.327118763

209 227265_at 7.302302834

210 238590_x_at 7.293895347

211 219690_at 7.292543061

212 1554966_a_at 7.278012354

213 1557169_x_at 7.268662905

214 218259_at 7.255132689

215 214452_at 7.238230245

216 227176_at 7.229027106

217 236562_at 7.226895333

218 218974_at 7.20994373

219 212135_s_at 7.200731635

220 208858_s_at 7.17415542

221 228095_at 7.163243578

222 238429_at 7.126375483

223 41660_at 7.124434474

224 204362_at 7.10915702

225 235953_at 7.105538551

226 227626_at 7.103123159

227 230986_at 7.089028843

228 228494_at 7.051103219

229 226587_at 7.043761204

230 212503_s_at 7.042735982

231 219302_s_at 7.031400154

232 231931_at 7.028235337

233 1559618_at 7.011096053

234 215554_at 6.986333437

235 220390_at 6.982804105

236 209268_at 6.979233016

237 213839_at 6.955891841

238 1562031_at 6.951840587

239 232914_s_at 6.946467462

240 232618_at 6.943559856

241 236694_at 6.940088211

242 235625_at 6.912044473

243 226683_at 6.910849206

244 238149_at 6.884748721

245 203798_s_at 6.870256677

246 238992_at 6.861290738

247 229234_at 6.856767006

248 203148_s_at 6.856726608

249 212653_s_at 6.843863568

250 226020_s_at 6.831865156

251 222192_s_at 6.825066013

252 202283_at 6.817836489

253 225988_at 6.776857803

254 1556467_at 6.769989048

255 203275_at 6.740821862

256 202869_at 6.736961244

257 204135_at 6.730380176

258 207761_s_at 6.729964991

259 205841_at 6.724409109

260 1557122_s_at 6.720215513

261 209707_at 6.705711893

262 215749_s_at 6.697160299

263 228055_at 6.691742819

264 221865_at 6.687068983

265 226485_at 6.664298399

266 242927_at 6.663113644

267 213906_at 6.660444675

268 231323_at 6.659648347

269 210173_at 6.603019928

270 208055_s_at 6.588684752

271 227157_at 6.586709852

272 213888_s_at 6.576878416

273 203656_at 6.569517886

274 219737_s_at 6.550940295

275 226338_at 6.54889046

276 228476_at 6.547208849

277 203046_s_at 6.543431904

278 228056_s_at 6.539836192

279 242512_at 6.511559021

280 233087_at 6.502207066

281 210347_s_at 6.498375003

282 223645_s_at 6.49116757

283 201528_at 6.48762756

284 225144_at 6.477257056

285 227156_at 6.445748954

286 211057_at 6.439697855

287 220338_at 6.424058363

288 204226_at 6.418225595

289 224839_s_at 6.410522718

290 213326_at 6.393300919

291 203881_s_at 6.38963712

292 238759_at 6.37018601

293 227606_s_at 6.36860988

294 228647_at 6.357193492

295 209829_at 6.322017374

296 235940_at 6.307994927

297 1558956_s_at 6.302752451

298 1552733_at 6.296084921

299 227822_at 6.269548276

300 229566_at 6.267855847

301 201529_s_at 6.243443714

302 221602_s_at 6.239947972

303 232353_s_at 6.237297186

304 224048_at 6.22258486

305 206167_s_at 6.219837979

306 1557167_at 6.217297009

307 228837_at 6.179945006

308 1553764_a_at 6.175797721

309 223422_s_at 6.157974956

310 210051_at 6.147553989

311 231270_at 6.141555075

312 213397_x_at 6.133971655

313 216733_s_at 6.126656641

314 225386_s_at 6.123920271

315 226041_at 6.105872652

316 220068_at 6.093850048

317 226510_at 6.085990155

318 211192_s_at 6.083647236

319 213677_s_at 6.07378988

320 1558186_s_at 6.055694813

321 206546_at 6.052440536

322 225847_at 6.036099133

323 239043_at 6.035586627

324 213689_x_at 6.031918768

325 225998_at 6.029396058

326 223178_s_at 6.024124139

327 241328_at 6.022975958

328 227639_at 6.022725474

329 209702_at 6.004370495

330 203607_at 6.0011334

331 219279_at 5.9990664

332 205140_at 5.995865413

333 214833_at 5.989534987

334 203829_at 5.988169257

335 239317_at 5.96304614

336 1552924_a_at 5.946737937

337 220066_at 5.94108529

338 226482_s_at 5.937855561

339 227945_at 5.936071626

340 205260_s_at 5.932340883

341 205484_at 5.901650558

342 202214_s_at 5.89643516

343 1563498_s_at 5.888913571

344 228195_at 5.847573134

345 216899_s_at 5.84220912

346 226068_at 5.826485819

347 230370_x_at 5.824325562

348 222154_s_at 5.822442552

349 241834_at 5.815865882

350 240239_at 5.79660629

351 202213_s_at 5.791471679

352 229937_x_at 5.75596971

353 240265_at 5.748202952

354 202502_at 5.743103255

355 230224_at 5.739682762

356 227568_at 5.731463219

357 205225_at 5.727279457

358 225384_at 5.708240026

359 232038_at 5.703879485

360 223646_s_at 5.69809431

361 203404_at 5.693189022

362 204834_at 5.678529969

363 206624_at 5.6740761

364 222357_at 5.668941966

365 208983_s_at 5.660808031

366 1557411_s_at 5.658060096

367 1558692_at 5.655268414

368 203427_at 5.646463266

369 213698_at 5.630876464

370 208923_at 5.620895525

371 214129_at 5.610506426

372 225485_at 5.600414079

373 205078_at 5.594053301

374 226721_at 5.585558334

375 227182_at 5.582533487

376 219924_s_at 5.566733432

377 211395_x_at 5.559996597

378 239824_s_at 5.557671257

379 231788_at 5.555360367

380 222427_s_at 5.546997211

381 206478_at 5.546628114

382 214130_s_at 5.543939447

383 222426_at 5.5416746

384 205410_s_at 5.540505079

385 223155_at 5.539308928

386 220751_s_at 5.537305042

387 206600_s_at 5.536228541

388 229974_at 5.535917718

389 221601_s_at 5.530922013

390 226603_at 5.529946412

391 215891_s_at 5.529177935

392 214720_x_at 5.521690405

393 226019_at 5.519742631

394 216748_at 5.5147577

395 204604_at 5.512695826

396 209421_at 5.511158044

397 201847_at 5.490711084

398 218491_s_at 5.472547228

399 225802_at 5.460408935

400 227020_at 5.457786652

401 206485_at 5.448235254

402 216765_at 5.442754637

403 1558722_at 5.442094467

404 1563876_at 5.433866042

405 212631_at 5.433308633

406 212538_at 5.431858885

407 228033_at 5.426513224

408 225274_at 5.42613898

409 209473_at 5.42524767

410 209782_s_at 5.4120625

411 239556_at 5.40849801

412 1559590_at 5.405349863

413 215145_s_at 5.394678561

414 238662_at 5.389378829

415 229285_at 5.379689847

416 229596_at 5.362779739

417 243747_at 5.349017783

418 229779_at 5.347149451

419 204688_at 5.346119181

420 218918_at 5.339530897

421 214835_s_at 5.33796372

422 238699_s_at 5.327875979

423 228654_at 5.324354019

424 208982_at 5.321039082

425 228174_at 5.319219236

426 226073_at 5.317401854

427 223467_at 5.317025922

428 238504_at 5.314050728

429 203551_s_at 5.308296662

430 218487_at 5.285078865

431 1555383_a_at 5.272283687

432 225484_at 5.25804504

433 204972_at 5.248528503

434 216044_x_at 5.242262149

435 205988_at 5.240262201

436 228585_at 5.236211966

437 1553991_s_at 5.232868482

438 226673_at 5.229646753

439 207100_s_at 5.221989294

440 229584_at 5.220199705

441 244023_at 5.214287027

442 212459_x_at 5.209343246

443 225579_at 5.208220395

444 212632_at 5.206024135

445 1561660_at 5.193675941

446 226119_at 5.18020072

447 214319_at 5.178025249

448 226344_at 5.175701806

449 204401_at 5.171450544

450 206395_at 5.161716838

451 227947_at 5.149493368

452 221103_s_at 5.145154859

453 236854_at 5.142794477

454 202761_s_at 5.139628204

455 221081_s_at 5.134732074

456 231727_s_at 5.133102254

457 1558826_at 5.132445846

458 223459_s_at 5.130578477

459 1555613_a_at 5.130541136

460 217286_s_at 5.117767119

461 224175_s_at 5.110987277

462 211675_s_at 5.105633571

463 228492_at 5.096350493

464 235522_at 5.091966755

465 207540_s_at 5.085374423

466 221211_s_at 5.076166884

467 219834_at 5.064925329

468 202139_at 5.062384192

469 203401_at 5.060666839

470 203343_at 5.058103297

471 215436_at 5.050737279

472 219573_at 5.049676616

473 201058_s_at 5.041716512

474 208981_at 5.034133585

475 208165_s_at 5.032638601

476 205842_s_at 5.026215401

477 201853_s_at 5.023831232

478 204045_at 5.020461541

479 227001_at 5.018876688

480 203763_at 5.017125408

481 227871_at 5.013676808

482 210144_at 5.010836624

483 214861_at 5.009648914

484 1552667_a_at 5.007529447

485 230032_at 5.00680407

486 32042_at 5.003199569

487 223269_at 4.994886861

488 226596_x_at 4.986546488

489 220992_s_at 4.984393609

490 218102_at 4.981193655

491 1554770_x_at 4.976867574

492 205053_at 4.974936116

493 204839_at 4.973317373

494 225346_at 4.969974674

495 214259_s_at 4.948375564

496 208151_x_at 4.94369418

497 230110_at 4.937189432

498 1558827_a_at 4.933033211

499 223272_s_at 4.929386147

500 217599_s_at 4.927384315

501 238447_at 4.923495706

502 221096_s_at 4.921179023

503 232014_at 4.915652733

504 225798_at 4.91141932

505 227139_s_at 4.910132649

506 211089_s_at 4.89839291

507 225017_at 4.889290071

508 239964_at 4.884214295

509 211883_x_at 4.882755371

510 221868_at 4.879301052

511 224722_at 4.878714297

512 226785_at 4.875600776

513 210036_s_at 4.861191607

514 65630_at 4.856395776

515 225112_at 4.855025927

516 219139_s_at 4.849479804

517 230788_at 4.842699556

518 239828_at 4.835141129

519 213587_s_at 4.828533209

520 228482_at 4.825762784

521 226665_at 4.823729475

522 238907_at 4.823525522

523 208719_s_at 4.821688666

524 213391_at 4.82151822

525 227247_at 4.819331363

526 226098_at 4.811260785

527 202053_s_at 4.810590514

528 218831_s_at 4.808535267

529 1562338_at 4.806460908

530 230803_s_at 4.802133463

531 224990_at 4.797863123

532 215772_x_at 4.797081665

533 213083_at 4.791860802

534 1554479_a_at 4.790092448

535 203498_at 4.781169326

536 221268_s_at 4.780939005

537 228788_at 4.779734556

538 227172_at 4.7791664

539 33646_g_at 4.773998126

540 230329_s_at 4.771545071

541 231956_at 4.767320107

542 227426_at 4.763349807

543 1555465_at 4.760133384

544 219283_at 4.758581235

545 211149_at 4.755051611

546 227412_at 4.754710492

547 203482_at 4.75270712

548 227785_at 4.751542719

549 204521_at 4.742596075

550 228730_s_at 4.739364101

551 204565_at 4.734663845

552 227478_at 4.733098677

553 218986_s_at 4.731858722

554 219185_at 4.730491212

555 212392_s_at 4.730469899

556 226948_at 4.724150742

557 242515_x_at 4.723703789

558 218981_at 4.723569548

559 231743_at 4.722058779

560 236994_at 4.716053223

561 218935_at 4.714479501

562 1553726_s_at 4.707191348

563 207112_s_at 4.702122094

564 229126_at 4.70201291

565 226864_at 4.694093244

566 212092_at 4.693702817

567 202983_at 4.690769143

568 224518_s_at 4.688540842

569 214484_s_at 4.688058264

570 235335_at 4.687665089

571 226496_at 4.686986047

572 212914_at 4.683856901

573 202330_s_at 4.67841227

574 212335_at 4.677467081

575 235512_at 4.66497387

576 224812_at 4.664703879

577 224735_at 4.660190724

578 223643_at 4.659825725

579 209269_s_at 4.655983676

580 225046_at 4.646015605

581 1570523_s_at 4.64259361

582 242774_at 4.632569113

583 205514_at 4.628818518

584 204724_s_at 4.626072897

585 213582_at 4.625624002

586 219080_s_at 4.624559653

587 214769_at 4.621462575

588 201135_at 4.620839564

589 238148_s_at 4.619648231

590 1556423_at 4.619454508

591 220146_at 4.617010457

592 223530_at 4.611595582

593 1558214_s_at 4.611069785

594 206752_s_at 4.608935251

595 203428_s_at 4.602339785

596 224406_s_at 4.599149198

597 205790_at 4.597624047

598 204992_s_at 4.596895914

599 212957_s_at 4.594842786

600 213958_at 4.594839601

601 207655_s_at 4.589283719

602 206700_s_at 4.583410493

603 226688_at 4.581547574

604 205037_at 4.580417169

605 1553400_a_at 4.577622519

606 204643_s_at 4.5709879

607 203803_at 4.568447576

608 238578_at 4.567018077

609 236918_s_at 4.564252166

610 226334_s_at 4.558810687

611 204731_at 4.55651242

612 1562089_at 4.556165017

613 1555571_at 4.541859255

614 221011_s_at 4.540458533

615 204319_s_at 4.530197664

616 240070_at 4.522810343

617 223391_at 4.517845704

618 210544_s_at 4.509300017

619 213626_at 4.508538996

620 225098_at 4.506461296

621 226258_at 4.502948569

622 200800_s_at 4.502271319

623 230036_at 4.502219827

624 207099_s_at 4.50159573

625 223681_s_at 4.495593219

626 201284_s_at 4.49531122

627 203263_s_at 4.494786219

628 212890_at 4.494736371

629 204547_at 4.491699771

630 229310_at 4.491123827

631 212609_s_at 4.490348755

632 221213_s_at 4.485809953

633 214830_at 4.479195078

634 214264_s_at 4.47863626

635 230619_at 4.476156568

636 212737_at 4.472684515

637 228855_at 4.4708666

638 230152_at 4.465838254

639 212791_at 4.463042361

640 207394_at 4.462698991

641 209509_s_at 4.462601553

642 1557113_at 4.459229649

643 241803_s_at 4.455012563

644 202738_s_at 4.453717344

645 228291_s_at 4.45142269

646 219494_at 4.444370221

647 206049_at 4.442148122

648 204105_s_at 4.440021

649 204410_at 4.439022435

650 221588_x_at 4.422468908

651 226625_at 4.42046764

652 235408_x_at 4.419210033

653 235478_at 4.412595493

654 219961_s_at 4.411983819

655 224893_at 4.410543664

656 219929_s_at 4.401274949

657 210006_at 4.399110987

658 204079_at 4.398107904

659 217838_s_at 4.393605998

660 231747_at 4.391535603

661 218545_at 4.387190949

662 202739_s_at 4.38336252

663 227839_at 4.379924496

664 219842_at 4.378855978

665 223206_s_at 4.373849259

666 1565681_s_at 4.370191502

667 238322_s_at 4.36921167

668 219641_at 4.366072237

669 203549_s_at 4.361919062

670 221267_s_at 4.361695332

671 205052_at 4.357143112

672 239533_at 4.357128012

673 230738_at 4.35287925

674 244774_at 4.352772141

675 207419_s_at 4.352692189

676 202923_s_at 4.349377706

677 224495_at 4.343885272

678 224802_at 4.34293542

679 212936_at 4.332343095

680 225501_at 4.330814862

681 1552296_at 4.330125983

682 231873_at 4.328516023

683 223337_at 4.326746209

684 224840_at 4.318193822

685 203144_s_at 4.317887035

686 201204_s_at 4.315521771

687 1554733_at 4.304448778

688 222557_at 4.304442811

689 203457_at 4.298625718

690 230712_at 4.297331285

691 214221_at 4.294981747

692 213018_at 4.294645353

693 202621_at 4.288486265

694 211502_s_at 4.285917263

695 211272_s_at 4.2824963

696 219574_at 4.280519802

697 211189_x_at 4.279993187

698 217944_at 4.276050822

699 240061_at 4.275221001

700 223457_at 4.2750032

701 219147_s_at 4.274847635

702 1555037_a_at 4.270505939

703 216915_s_at 4.260379658

704 207691_x_at 4.255540849

705 218418_s_at 4.249582174

706 203284_s_at 4.245764937

707 221044_s_at 4.242920068

708 235714_at 4.23979497

709 221909_at 4.238860534

710 203940_s_at 4.238331699

711 224759_s_at 4.23791896

712 235010_at 4.224155663

713 212946_at 4.222497298

714 230925_at 4.215392592

715 221082_s_at 4.212341785

716 235085_at 4.20626866

717 209474_s_at 4.204414771

718 204354_at 4.202287886

719 202006_at 4.195125586

720 204867_at 4.193007759

721 219307_at 4.189997839

722 210787_s_at 4.18791163

723 217104_at 4.187061185

724 221185_s_at 4.182065043

725 201276_at 4.175599953

726 215997_s_at 4.174676772

727 204308_s_at 4.174466987

728 225763_at 4.171091614

729 227350_at 4.170458494

730 232820_s_at 4.164678129

731 207777_s_at 4.15247505

732 218248_at 4.14964523

733 220477_s_at 4.149394998

734 210257_x_at 4.146856143

735 210189_at 4.146044209

736 224404_s_at 4.145255421

737 220999_s_at 4.14078415

738 230753_at 4.135952165

739 228841_at 4.13434419

740 229954_at 4.132832804

741 233198_at 4.121032953

742 209894_at 4.119532145

743 231984_at 4.119383665

744 203077_s_at 4.118398691

745 205628_at 4.115134258

746 219505_at 4.113119535

747 203820_s_at 4.112518019

748 213725_x_at 4.111705684

749 218440_at 4.109409211

750 1555874_x_at 4.108383906

751 210694_s_at 4.106993033

752 218756_s_at 4.106076482

753 212523_s_at 4.104137312

754 222880_at 4.10385

755 228909_at 4.101037683

756 224522_s_at 4.098190364

757 203814_s_at 4.098095203

758 1555363_s_at 4.095400376

759 212334_at 4.095205929

760 211208_s_at 4.093290335

761 212956_at 4.089495851

762 202342_s_at 4.087199034

763 226455_at 4.085598686

764 1552552_s_at 4.084427854

765 235643_at 4.08432452

766 220183_s_at 4.083083298

767 204290_s_at 4.082472026

768 218209_s_at 4.082276777

769 207766_at 4.080811298

770 222931_s_at 4.079243139

771 222553_x_at 4.079086215

772 213269_at 4.078821861

773 220341_s_at 4.076334662

774 218321_x_at 4.075786552

775 1559419_at 4.073560966

776 1554772_at 4.066575784

777 236917_at 4.065227243

778 227699_at 4.064363679

779 203454_s_at 4.063963656

780 227864_s_at 4.06287506

781 200703_at 4.057016022

782 226995_at 4.054203482

783 221666_s_at 4.05061088

784 221974_at 4.044070086

785 212912_at 4.042776645

786 231579_s_at 4.040031383

787 218513_at 4.038569872

788 226287_at 4.03808142

789 213058_at 4.035308578

790 232950_s_at 4.028770153

791 206316_s_at 4.028274509

792 202922_at 4.02248628

793 229588_at 4.015847543

794 237052_x_at 4.014731485

795 236539_at 4.012437722

796 224392_s_at 4.010524706

797 235729_at 4.006448689

798 223411_at 4.005515706
