# Supplementary material for: Hsp90 inhibition increases SOCS3 transcript and regulates migration and cell death in chronic lymphocytic leukemia
Source: Oncotarget. 2016 Apr 16;7(19):28684–96. doi: 10.18632/oncotarget.8760 (PMC5053755; doi:10.18632/oncotarget.8760)
Supplement: Supplementary file 3 [file oncotarget-07-28684-s003.docx]

Supplemental Table 2: Probe sets down-regulated 4-fold or greater in CLL vs NB cells

# Probe fold:CLL/Normal B

1 209189_at -97.04870117

2 207978_s_at -96.72859242

3 204790_at -88.43597746

4 225673_at -78.69930554

5 204014_at -76.29816738

6 209959_at -68.51274578

7 227697_at -60.60143286

8 225262_at -55.23475787

9 216979_at -52.27301615

10 221841_s_at -41.92020887

11 228188_at -40.57474155

12 216248_s_at -36.49891063

13 203411_s_at -35.97233739

14 205239_at -34.90973499

15 204621_s_at -34.64338506

16 201739_at -34.57629785

17 204015_s_at -33.58678968

18 202859_x_at -33.06207805

19 215176_x_at -31.42591836

20 204622_x_at -31.05004599

21 224920_x_at -30.15869295

22 225955_at -28.52127358

23 1559975_at -27.65849018

24 221704_s_at -27.52080948

25 201195_s_at -27.33480162

26 235048_at -26.28452029

27 230511_at -25.17944363

28 242904_x_at -24.77133128

29 209324_s_at -24.11167548

30 214446_at -23.70083529

31 224588_at -23.22900575

32 226099_at -22.68623823

33 202988_s_at -20.30755997

34 212813_at -20.12705937

35 234764_x_at -20.06895372

36 207630_s_at -18.98230128

37 202284_s_at -18.64683019

38 212975_at -18.57273017

39 227671_at -17.91923323

40 212592_at -17.74837456

41 205207_at -17.66297557

42 202672_s_at -17.59380351

43 226982_at -17.27089569

44 231721_at -17.26308022

45 215501_s_at -17.15575841

46 214508_x_at -16.68536105

47 206207_at -16.23779128

48 208092_s_at -16.20462804

49 224590_at -16.153143

50 221563_at -16.0062118

51 205885_s_at -15.87261032

52 203045_at -15.79030354

53 216576_x_at -15.77318937

54 209191_at -15.72801285

55 204472_at -15.70411241

56 209967_s_at -15.63590154

57 216236_s_at -15.43191824

58 211506_s_at -15.26403522

59 209683_at -14.50049254

60 216401_x_at -14.39181359

61 212274_at -14.13763112

62 218094_s_at -14.01674056

63 209116_x_at -13.95550088

64 218880_at -13.66023597

65 212124_at -13.62259834

66 213416_at -13.60362748

67 226682_at -13.42288611

68 220712_at -13.41249753

69 204567_s_at -13.25028972

70 227613_at -13.19459808

71 209301_at -13.18203773

72 1556361_s_at -13.16911052

73 202600_s_at -13.14910773

74 205884_at -13.08250586

75 202498_s_at -13.07702534

76 211458_s_at -12.89923408

77 216834_at -12.622432

78 228263_at -12.6062913

79 222088_s_at -12.43026803

80 201466_s_at -12.36184349

81 216207_x_at -12.16194445

82 205174_s_at -12.12979699

83 219396_s_at -12.09511013

84 203749_s_at -12.06414275

85 205590_at -11.97362012

86 219228_at -11.86507241

87 212276_at -11.79216736

88 217232_x_at -11.75956264

89 206683_at -11.71091584

90 202499_s_at -11.59758086

91 202497_x_at -11.51671213

92 224589_at -11.09947474

93 212272_at -11.05020324

94 209325_s_at -11.04702887

95 212086_x_at -10.97760554

96 209574_s_at -10.82516793

97 214470_at -10.77541105

98 1554999_at -10.62714749

99 214230_at -10.58225366

100 1568768_s_at -10.58066207

101 201670_s_at -10.55732553

102 211113_s_at -10.46329618

103 226430_at -10.43368588

104 202599_s_at -10.38866236

105 208869_s_at -10.33277962

106 210232_at -10.28048555

107 202464_s_at -10.23276057

108 211696_x_at -10.20168815

109 211745_x_at -10.14865405

110 228097_at -10.11629775

111 1556066_at -10.00195421

112 213146_at -9.983910491

113 243386_at -9.968894321

114 211699_x_at -9.928732119

115 222142_at -9.921989978

116 217414_x_at -9.863803092

117 201041_s_at -9.832893383

118 209458_x_at -9.816171808

119 216491_x_at -9.810254067

120 218854_at -9.761920482

121 201044_x_at -9.744448253

122 214414_x_at -9.710243675

123 60084_at -9.688978019

124 202431_s_at -9.659673993

125 209457_at -9.63593058

126 204018_x_at -9.535816672

127 211199_s_at -9.534838483

128 204995_at -9.174502958

129 1556067_a_at -9.13950164

130 1561042_at -9.05212182

131 225897_at -8.973762487

132 224847_at -8.836070932

133 210001_s_at -8.769855064

134 231720_s_at -8.767302339

135 214445_at -8.754313202

136 220266_s_at -8.695002848

137 213002_at -8.672127694

138 214218_s_at -8.59147597

139 230276_at -8.559642247

140 229450_at -8.478925797

141 211423_s_at -8.393587126

142 231592_at -8.375422678

143 213975_s_at -8.247855172

144 205883_at -8.187006269

145 209267_s_at -8.18040063

146 1566403_at -8.172630449

147 228789_at -8.168657533

148 201464_x_at -8.156860618

149 221728_x_at -8.145402554

150 228749_at -8.122486834

151 204435_at -8.110267503

152 1555938_x_at -8.078158762

153 227684_at -8.025462301

154 1558438_a_at -8.024914381

155 205398_s_at -7.906356455

156 220302_at -7.89980477

157 228964_at -7.889571793

158 219956_at -7.883550391

159 208868_s_at -7.744816193

160 214108_at -7.728856606

161 202147_s_at -7.728018244

162 213281_at -7.722931098

163 204430_s_at -7.711360957

164 1554095_at -7.690215357

165 211605_s_at -7.687057713

166 41386_i_at -7.685744409

167 201669_s_at -7.660563401

168 1568665_at -7.606817538

169 1555745_a_at -7.571919115

170 213138_at -7.511788795

171 206342_x_at -7.45049614

172 218881_s_at -7.423137025

173 206715_at -7.391732255

174 201473_at -7.361146583

175 203358_s_at -7.342419279

176 203504_s_at -7.288750416

177 202381_at -7.241487114

178 205624_at -7.241258734

179 225954_s_at -7.196552746

180 204735_at -7.178221792

181 203751_x_at -7.118407383

182 209239_at -7.101594574

183 201465_s_at -7.098700763

184 202308_at -7.067602983

185 214326_x_at -7.046053846

186 218284_at -7.032092267

187 210904_s_at -7.022890787

188 1554757_a_at -7.02273745

189 206115_at -6.977530376

190 200731_s_at -6.92072414

191 205249_at -6.903275206

192 224836_at -6.901246671

193 204440_at -6.827819879

194 223492_s_at -6.825953091

195 241027_at -6.815393707

196 200730_s_at -6.810598101

197 210793_s_at -6.803445207

198 202643_s_at -6.789260774

199 225827_at -6.78454234

200 213310_at -6.774535356

201 226499_at -6.768184985

202 202684_s_at -6.752775055

203 202638_s_at -6.749838575

204 208078_s_at -6.743649246

205 204447_at -6.707555097

206 217227_x_at -6.706337085

207 208949_s_at -6.680659364

208 219869_s_at -6.671032177

209 202014_at -6.633967756

210 227404_s_at -6.625654615

211 203505_at -6.621003991

212 222044_at -6.615162058

213 226026_at -6.588753256

214 227337_at -6.587723484

215 205020_s_at -6.570512932

216 1565358_at -6.568814386

217 223028_s_at -6.502986821

218 205397_x_at -6.501093935

219 215889_at -6.401198291

220 1554036_at -6.361755826

221 1555630_a_at -6.360212641

222 205114_s_at -6.301553347

223 206675_s_at -6.299909046

224 216260_at -6.291168486

225 202146_at -6.249737087

226 218696_at -6.220139775

227 230345_at -6.219801333

228 204249_s_at -6.218941301

229 216412_x_at -6.212967467

230 200732_s_at -6.199895934

231 244700_at -6.176523348

232 219312_s_at -6.162240547

233 1564093_at -6.143626455

234 232379_at -6.133691046

235 217378_x_at -6.12760797

236 37028_at -6.124104921

237 201668_x_at -6.098054313

238 201888_s_at -6.074465628

239 222309_at -6.004738835

240 214429_at -5.950312755

241 231990_at -5.925091773

242 241985_at -5.922677373

243 202887_s_at -5.914175273

244 206919_at -5.901454207

245 243372_at -5.890538385

246 221773_at -5.888201326

247 201212_at -5.859325104

248 205396_at -5.857962671

249 222343_at -5.823336553

250 217258_x_at -5.767867407

251 1564150_a_at -5.75924821

252 211612_s_at -5.752376081

253 202861_at -5.747033664

254 241425_at -5.746952002

255 242218_at -5.743262489

256 1564027_a_at -5.716715522

257 228953_at -5.694189476

258 233899_x_at -5.691959908

259 1554309_at -5.691214282

260 222838_at -5.677307955

261 225140_at -5.676487523

262 1555392_at -5.674980755

263 201861_s_at -5.672361584

264 207535_s_at -5.6722397

265 202531_at -5.671946797

266 205681_at -5.661237701

267 213241_at -5.657138531

268 203006_at -5.653036453

269 1555689_at -5.652713195

270 41387_r_at -5.637499889

271 238725_at -5.586960034

272 224654_at -5.583769934

273 219957_at -5.562430865

274 202637_s_at -5.558775046

275 215064_at -5.534595959

276 239401_at -5.533337799

277 208536_s_at -5.512701557

278 203574_at -5.507238164

279 1562255_at -5.506114076

280 240777_at -5.489674082

281 216504_s_at -5.454235525

282 202391_at -5.423301963

283 227410_at -5.421242341

284 218532_s_at -5.4179835

285 201829_at -5.409178476

286 209795_at -5.376107551

287 235574_at -5.374043503

288 1555372_at -5.348615517

289 226039_at -5.330986394

290 1555847_a_at -5.324473964

291 203408_s_at -5.317291283

292 201694_s_at -5.31411703

293 214583_at -5.294360745

294 238840_at -5.266191556

295 215707_s_at -5.262602763

296 228098_s_at -5.255913382

297 206127_at -5.249807416

298 217739_s_at -5.243879376

299 219657_s_at -5.243099774

300 220308_at -5.240959644

301 207275_s_at -5.224152458

302 202644_s_at -5.220642974

303 210845_s_at -5.189555597

304 209558_s_at -5.187996477

305 203616_at -5.177840412

306 223130_s_at -5.161443141

307 1553861_at -5.152906804

308 1554929_at -5.124641206

309 201329_s_at -5.113896634

310 217480_x_at -5.111467331

311 216853_x_at -5.108504471

312 209636_at -5.106318405

313 223343_at -5.104814369

314 205214_at -5.066461511

315 209684_at -5.049960138

316 235567_at -5.04067172

317 225133_at -5.034430192

318 235020_at -5.025037978

319 205027_s_at -5.01508273

320 216300_x_at -4.994242935

321 208152_s_at -4.982120791

322 201939_at -4.981471605

323 235421_at -4.977053862

324 221658_s_at -4.889085041

325 206147_x_at -4.88684044

326 1553096_s_at -4.868532592

327 227999_at -4.856427755

328 213758_at -4.852783508

329 203936_s_at -4.838207015

330 202671_s_at -4.833510917

331 205119_s_at -4.821324387

332 207945_s_at -4.819114235

333 217591_at -4.808368619

334 221140_s_at -4.797843169

335 211637_x_at -4.789883277

336 224923_at -4.788595254

337 223129_x_at -4.785884234

338 1557267_s_at -4.784645375

339 224710_at -4.7758764

340 209305_s_at -4.767577861

341 207624_s_at -4.762169594

342 218360_at -4.758089799

343 204639_at -4.756266324

344 238534_at -4.728519348

345 201543_s_at -4.727652513

346 220319_s_at -4.723600652

347 201963_at -4.690240821

348 238488_at -4.684528996

349 204103_at -4.683804956

350 201300_s_at -4.680270779

351 238900_at -4.674546795

352 212758_s_at -4.665813042

353 204439_at -4.665657808

354 201887_at -4.657061713

355 217992_s_at -4.641856747

356 243296_at -4.637711254

357 202340_x_at -4.618436397

358 239930_at -4.617346498

359 1555963_x_at -4.594497237

360 212666_at -4.579675889

361 205691_at -4.575987147

362 216226_at -4.574227119

363 225615_at -4.563844067

364 1555279_at -4.562433402

365 224783_at -4.559025567

366 231911_at -4.54990682

367 209573_s_at -4.535097288

368 206359_at -4.529285558

369 235199_at -4.525940128

370 203907_s_at -4.523047039

371 224978_s_at -4.521810397

372 211899_s_at -4.518987292

373 205692_s_at -4.514117589

374 209304_x_at -4.513798448

375 1552532_a_at -4.501739265

376 207574_s_at -4.482714132

377 208960_s_at -4.465330624

378 1560225_at -4.464041688

379 223887_at -4.455957585

380 209163_at -4.45430084

381 1558143_a_at -4.447256142

382 38340_at -4.445211306

383 225569_at -4.444841579

384 226487_at -4.439434758

385 1553785_at -4.438304038

386 207996_s_at -4.425570669

387 200733_s_at -4.419599072

388 208937_s_at -4.418514749

389 235175_at -4.4036399

390 225864_at -4.402817363

391 224454_at -4.398799976

392 213450_s_at -4.392843187

393 1554519_at -4.391692371

394 210875_s_at -4.390105165

395 239379_at -4.387419028

396 201862_s_at -4.386588879

397 1555167_s_at -4.382952367

398 201360_at -4.375037855

399 1554557_at -4.373926569

400 218928_s_at -4.370470196

401 223027_at -4.370252086

402 234366_x_at -4.362232

403 214467_at -4.360783903

404 211197_s_at -4.348446246

405 201718_s_at -4.333134445

406 230052_s_at -4.332725988

407 208488_s_at -4.331709518

408 217157_x_at -4.325740137

409 228562_at -4.321951851

410 215375_x_at -4.321766119

411 209711_at -4.317894518

412 224450_s_at -4.317628154

413 36711_at -4.314069743

414 214704_at -4.312766176

415 214211_at -4.310702502

416 220306_at -4.308500944

417 204429_s_at -4.294899878

418 1555370_a_at -4.279800358

419 225710_at -4.274468375

420 229865_at -4.267109108

421 227513_s_at -4.263107691

422 201502_s_at -4.251037542

423 229452_at -4.249867905

424 206374_at -4.244518785

425 227723_at -4.22710808

426 36829_at -4.219997078

427 229763_at -4.219555414

428 1568695_s_at -4.215602973

429 214696_at -4.209427401

430 220953_s_at -4.204894197

431 217996_at -4.188650468

432 209993_at -4.185368064

433 244828_x_at -4.180876709

434 1561908_a_at -4.179904556

435 212226_s_at -4.174132798

436 205281_s_at -4.171617842

437 201490_s_at -4.166328225

438 209184_s_at -4.165203548

439 1555281_x_at -4.161131843

440 215214_at -4.160464186

441 242288_s_at -4.159096042

442 212636_at -4.155290959

443 1553042_a_at -4.148397097

444 211676_s_at -4.142832524

445 201751_at -4.136856747

446 214157_at -4.122258566

447 201445_at -4.115122849

448 228976_at -4.106585968

449 223780_s_at -4.106092135

450 209999_x_at -4.100833019

451 206118_at -4.098565346

452 213805_at -4.093183939

453 218510_x_at -4.086505001

454 204794_at -4.079834133

455 230707_at -4.077931383

456 209276_s_at -4.077190879

457 235568_at -4.052505096

458 227680_at -4.044449928

459 218898_at -4.042853708

460 227953_at -4.032857697

461 206983_at -4.024113509

462 203321_s_at -4.01170633

463 203085_s_at -4.010931979

464 212842_x_at -4.010073

465 219500_at -4.008543138

466 212764_at -4.003855755

467 224924_at -4.00234075
