# Supplementary material for: Hsp90 inhibition increases SOCS3 transcript and regulates migration and cell death in chronic lymphocytic leukemia
Source: Oncotarget. 2016 Apr 16;7(19):28684–96. doi: 10.18632/oncotarget.8760 (PMC5053755; doi:10.18632/oncotarget.8760)
Supplement: Supplementary file 4 [file oncotarget-07-28684-s004.docx]

Supplemental Table 3: Probe sets down-regulated 4-fold or greater in DMAG treated vs Vehicle

# Probe fold:CLL DMAG/CLL Vehicle

1 206641_at -24.24810333

2 206478_at -20.78109832

3 220068_at -20.16665637

4 1567628_at -16.24247973

5 206115_at -15.30042689

6 219371_s_at -12.48219716

7 205901_at -11.36164637

8 227404_s_at -11.04713225

9 228055_at -10.83930236

10 209619_at -10.32378431

11 223750_s_at -10.00465838

12 219301_s_at -9.981108155

13 204401_at -9.850520784

14 222435_s_at -9.766895088

15 217826_s_at -9.69069408

16 215145_s_at -9.682636939

17 219302_s_at -9.660514325

18 228056_s_at -9.315285588

19 242020_s_at -9.272767615

20 203932_at -8.950076415

21 203290_at -8.946975092

22 206206_at -8.887637656

23 212592_at -8.8802482

24 221491_x_at -8.665001505

25 205671_s_at -8.635621432

26 217823_s_at -8.61111467

27 206983_at -8.440918532

28 214369_s_at -8.425720154

29 201170_s_at -8.415214234

30 201850_at -8.116175154

31 213620_s_at -8.048388799

32 205306_x_at -7.89369075

33 217824_at -7.841338824

34 205307_s_at -7.802302859

35 202081_at -7.652334827

36 211138_s_at -7.495371575

37 219300_s_at -7.489658818

38 208206_s_at -7.427105132

39 206513_at -7.294959659

40 242344_at -7.131472531

41 213831_at -7.105814367

42 210982_s_at -7.093019924

43 223595_at -7.082211852

44 206687_s_at -7.035729416

45 207826_s_at -6.724840266

46 205249_at -6.61939792

47 219183_s_at -6.603358625

48 221239_s_at -6.562747833

49 213674_x_at -6.514254119

50 217825_s_at -6.493066567

51 210448_s_at -6.403230747

52 227189_at -6.399681027

53 236203_at -6.342273093

54 238900_at -6.26710863

55 244261_at -6.251489622

56 224735_at -6.248890241

57 208438_s_at -6.225975933

58 208121_s_at -6.140261083

59 220377_at -6.127506035

60 215118_s_at -6.12113845

61 205789_at -6.120289939

62 211986_at -6.110964076

63 205861_at -6.109269992

64 224193_s_at -6.091088221

65 212671_s_at -6.017232074

66 203761_at -6.00431639

67 205718_at -5.961601738

68 203760_s_at -5.938917534

69 209498_at -5.915089509

70 1563674_at -5.867719882

71 223751_x_at -5.82718858

72 204683_at -5.799385335

73 211430_s_at -5.773715418

74 208894_at -5.75653229

75 212998_x_at -5.651367467

76 244623_at -5.575495405

77 209823_x_at -5.552740596

78 202759_s_at -5.517826141

79 235281_x_at -5.509800186

80 226147_s_at -5.491118327

81 223423_at -5.373641218

82 208763_s_at -5.365081177

83 202760_s_at -5.29672277

84 1554240_a_at -5.271450535

85 209480_at -5.269989181

86 206082_at -5.240120544

87 226694_at -5.234675113

88 212099_at -5.23104797

89 212999_x_at -5.228148065

90 205552_s_at -5.212227301

91 230047_at -5.206089089

92 211656_x_at -5.189156332

93 215666_at -5.188437012

94 212827_at -5.179094921

95 220146_at -5.119489655

96 226272_at -5.119489655

97 1556423_at -5.108855037

98 217281_x_at -5.100009732

99 222062_at -5.088004688

100 203186_s_at -5.072862301

101 214916_x_at -5.06092116

102 205180_s_at -4.955394791

103 205114_s_at -4.942017091

104 212750_at -4.934486635

105 207583_at -4.922871221

106 211644_x_at -4.908560509

107 216191_s_at -4.8966667

108 211654_x_at -4.892595461

109 233813_at -4.889883182

110 202207_at -4.823231311

111 206170_at -4.819555182

112 202206_at -4.819555182

113 1553196_a_at -4.813545742

114 1556467_at -4.796227195

115 209374_s_at -4.780627509

116 211991_s_at -4.77599061

117 205659_at -4.709258771

118 216557_x_at -4.693291276

119 1557122_s_at -4.663780856

120 215925_s_at -4.653447723

121 215193_x_at -4.65183524

122 228518_at -4.6476454

123 242157_at -4.626432045

124 1558185_at -4.594156491

125 217478_s_at -4.575406739

126 217022_s_at -4.574772497

127 205859_at -4.568434909

128 211634_x_at -4.551682731

129 236832_at -4.540968409

130 227134_at -4.537507413

131 203729_at -4.521495413

132 214995_s_at -4.497427422

133 204670_x_at -4.485907897

134 229390_at -4.485596969

135 242520_s_at -4.469458341

136 211742_s_at -4.466051848

137 210512_s_at -4.46202934

138 204205_at -4.458319471

139 214971_s_at -4.455539094

140 204642_at -4.45399519

141 52164_at -4.450292001

142 224606_at -4.44474299

143 202869_at -4.443818828

144 214933_at -4.44258691

145 41577_at -4.437662655

146 238480_at -4.43151501

147 215621_s_at -4.424149078

148 235085_at -4.393589248

149 202478_at -4.384158631

150 202687_s_at -4.36747668

151 202688_at -4.355384235

152 234440_at -4.346035623

153 208498_s_at -4.318110014

154 230983_at -4.297804922

155 205297_s_at -4.294826943

156 217591_at -4.287688209

157 241844_x_at -4.283529428

158 213060_s_at -4.279671316

159 231093_at -4.278188352

160 225033_at -4.271373333

161 219014_at -4.261318838

162 242541_at -4.248931249

163 207761_s_at -4.247753359

164 229151_at -4.245987135

165 211965_at -4.242750965

166 221756_at -4.220166736

167 208147_s_at -4.209357375

168 1562754_at -4.201195726

169 220131_at -4.194212624

170 201506_at -4.173332881

171 206707_x_at -4.142208

172 220132_s_at -4.134463118

173 210895_s_at -4.128163182

174 220390_at -4.126446684

175 213280_at -4.114736368

176 235146_at -4.103343698

177 219452_at -4.103059285

178 50221_at -4.086313808

179 213888_s_at -4.083199337

180 206675_s_at -4.061464403

181 215949_x_at -4.056681395

182 215275_at -4.054994619

183 201169_s_at -4.054713558

184 222952_s_at -4.052184885

185 222285_at -4.051904019

186 1556839_s_at -4.050780748

187 233500_x_at -4.041805786

188 1555832_s_at -4.006104348

189 214329_x_at -4.00360599
