# Supplementary material for: Hsp90 inhibition increases SOCS3 transcript and regulates migration and cell death in chronic lymphocytic leukemia
Source: Oncotarget. 2016 Apr 16;7(19):28684–96. doi: 10.18632/oncotarget.8760 (PMC5053755; doi:10.18632/oncotarget.8760)
Supplement: Supplementary file 5 [file oncotarget-07-28684-s005.docx]

Supplemental Table 4: Probe sets up-regulated 4-fold or greater in DMAG treated vs Vehicle

# Probe fold:CLL DMAG/CLL Vehicle

1 228188_at 18.14122534

2 219727_at 18.08723532

3 241716_at 16.83976383

4 225262_at 13.67813852

5 226430_at 12.88392266

6 227613_at 12.40371175

7 219228_at 10.34886037

8 207848_at 9.938306254

9 204627_s_at 9.855301446

10 219159_s_at 9.056791217

11 222838_at 9.048006715

12 227697_at 8.960007873

13 204014_at 8.955661505

14 209574_s_at 8.781089798

15 209959_at 8.654797112

16 200800_s_at 8.522046952

17 205133_s_at 8.335102843

18 207978_s_at 8.297633869

19 243372_at 8.17716074

20 216248_s_at 8.140966036

21 209967_s_at 7.99390263

22 230511_at 7.910670603

23 207630_s_at 7.725883912

24 202581_at 7.614239767

25 214508_x_at 7.332473744

26 204622_x_at 7.266698252

27 202393_s_at 7.140870722

28 200806_s_at 6.991490242

29 208744_x_at 6.935981257

30 221563_at 6.815880303

31 1554333_at 6.262766106

32 200799_at 6.078014009

33 204621_s_at 6.02725039

34 204798_at 6.007646813

35 215501_s_at 5.934391062

36 239436_at 5.785332991

37 206976_s_at 5.751347465

38 208575_at 5.7394003

39 217911_s_at 5.605721224

40 225955_at 5.543895256

41 211458_s_at 5.538518036

42 210370_s_at 5.279495227

43 200894_s_at 5.274740062

44 200895_s_at 5.274008879

45 203835_at 5.183045289

46 201641_at 5.159388196

47 200664_s_at 5.124815263

48 200666_s_at 5.092591512

49 205572_at 5.010657754

50 232914_s_at 4.952647699

51 220613_s_at 4.926967653

52 200807_s_at 4.925601797

53 225496_s_at 4.91468857

54 211968_s_at 4.905159333

55 1554334_a_at 4.886833665

56 202988_s_at 4.728229293

57 225061_at 4.696871096

58 208869_s_at 4.673489006

59 215967_s_at 4.659903245

60 231472_at 4.639598618

61 219888_at 4.598298111

62 203810_at 4.590973167

63 214554_at 4.547267893

64 224707_at 4.54033894

65 215688_at 4.489329536

66 213714_at 4.483110315

67 212009_s_at 4.469148552

68 214516_at 4.460483186

69 216979_at 4.292148526

70 203811_s_at 4.271373333

71 201841_s_at 4.268117815

72 216834_at 4.237754463

73 236034_at 4.236873339

74 219359_at 4.214612521

75 1559420_x_at 4.212860077

76 210550_s_at 4.204108783

77 214509_at 4.195375667

78 225408_at 4.195375667

79 208046_at 4.166396091

80 204916_at 4.068790504

81 213330_s_at 4.045449464

82 229974_at 4.028101397

83 214481_at 4.02307881

84 210691_s_at 4.019455282

85 205489_at 4.015278342

86 218566_s_at 4.014721745

87 201491_at 4.005271389
